# A Biodegradable Fish-Derived Gelatin–Chitosan Sponge as a Novel Hemostatic Agent in a Rat Tooth Extraction Model

**DOI:** 10.3390/md24100345

**Published:** 2026-09-30

**Authors:** Pruput D. Mutiari, Intan N. Dhewayani, Lilies D. Sulistyani, Lisa R. Amir, Arief Boediono, Vera Julia, Dwi Ariawan

**Affiliations:** 1 Department of Oral and Maxillofacial Surgery, Faculty of Dentistry, Universitas Indonesia, Jalan Salemba Raya 4 Central, Jakarta 10430, Indonesia; mutiari02@gmail.com (P.D.M.); dhewayaniintan@gmail.com (I.N.D.); vera.julia@ui.ac.id (V.J.);; 2 Department of Oral Biology, Faculty of Dentistry, Universitas Indonesia, Jakarta 10430, Indonesia; 3 School of Veterinary Medicine and Biomedical Sciences, Institut Pertanian Bogor University, Bogor 16680, Indonesia

**Keywords:** fish-derived gelatin, chitosan, hemostatic biomaterial, rat tooth

## Abstract

Background: Rapid hemostasis is essential in oral surgery to minimize blood loss, prevent postoperative complications, and promote early wound healing. This study developed freeze-dried fish-derived gelatin–chitosan composite sponges with different polymer compositions (G100, GC75, GC50, and GC25) and regularly investigated the relationships among scaffold microstructure, physicochemical properties, biological performance, and hemostatic efficacy. Methods: The sponges were characterized using Fourier transform infrared spectroscopy, scanning electron microscopy, mechanical testing, swelling, and degradation analyses. Biological performance was evaluated through cytocompatibility and antibacterial assays, while hemostatic function was assessed using BCI, BCT, fibrin generation, PT, and PTT. Results: Among the formulations, the gelatin-rich composite GC75 exhibits an interconnected porous structure, high fluid-absorption capacity, and a distinct degradation profile while maintaining acceptable cytocompatibility. Compared with the other formulations and a commercial gelatin sponge, GC75 demonstrated significantly enhanced hemostatic performance, as evidenced by lower BCI values, shorter BCT, and accelerated fibrin generation (all *p* < 0.001), while maintaining physiological PT and aPTT values. In a rat tooth extraction model, GC75 significantly reduced bleeding time and achieved hemostatic efficacy comparable to or better than the commercial control. Conclusions: These findings demonstrate the potential of gelatin-rich fish-derived gelatin–chitosan composite sponges as biodegradable hemostatic biomaterials for oral surgical applications.

## 1. Introduction

Hemostasis is the first and one of the most critical stages of wound healing, serving to rapidly arrest bleeding while establishing a provisional fibrin matrix that supports subsequent tissue repair [1]. In oral and maxillofacial surgery, including tooth extraction, periodontal surgery and implant placement, effective bleeding control is essential to maintain surgical visibility, minimize postoperative complications, and promote predictable healing [2]. Uncontrolled bleeding may prolong operative time, increase the risk of infection, impair tissue regeneration, and compromise patient recovery [3]. These challenges become even more pronounced in patients receiving anticoagulant therapy or presenting with coagulation disorders, highlighting the continuing clinical need for safe and effective topical hemostatic materials capable of rapidly stabilizing blood clots without interfering with normal tissue healing [2,4].

Various absorbable hemostatic agents have been developed for surgical applications, including collagen, oxidized regenerated cellulose, gelatin, alginate, chitosan, carboxymethyl cellulose, zeolite-based materials, and synthetic polymeric dressings [5,6]. Among these, porous sponge-type biomaterials have attracted considerable attention because their interconnected three-dimensional architecture enables rapid blood absorption, concentration of coagulation factors, platelet adhesion, and fibrin network formation, thereby accelerating clot stabilization at the wound site [7]. Compared with conventional surgical gauze, sponge-based materials exhibit greater fluid uptake capacity and better adaptability to irregular wound geometries, reducing postoperative complications such as hematoma and persistent bleeding [6,8].

Marine-derived biomaterials have recently emerged as attractive alternatives to conventional mammalian-derived biomaterials because of their excellent biocompatibility, abundant availability, low risk of transmitting zoonotic pathogens, and greater acceptance across diverse religious and cultural communities [5,6]. Furthermore, marine gelatin can be readily processed into highly porous sponge architectures through freeze-drying techniques, providing interconnected pore networks that facilitate rapid blood infiltration, platelet retention, and fibrin deposition during the early stages of hemostasis [5,7]. These structural and biological characteristics make fish-derived gelatin a promising candidate for the development of biodegradable topical hemostatic materials for oral surgical applications.

Despite these advantages, gelatin alone exhibits several intrinsic limitations, including relatively poor mechanical strength, rapid degradation under physiological conditions, and insufficient structural stability after blood absorption, which may compromise its clinical performance as a hemostatic scaffold [5]. To overcome these limitations, chitosan has been widely incorporated into gelatin-based biomaterials because of its excellent biocompatibility, biodegradability, antibacterial activity, and intrinsic hemostatic properties [8,9]. Unlike many conventional hemostatic agents that primarily depend on activation of the coagulation cascade, chitosan promotes hemostasis through electrostatic interactions between its positively charged amino groups and negatively charged erythrocyte membranes and platelets, thereby enhancing cellular aggregation, platelet adhesion, and fibrin network formation at the bleeding site [8,10]. In addition, incorporation of chitosan improves the mechanical integrity and degradation behavior of gelatin-based scaffolds, enabling the composite material to provide both structural support and enhanced blood–material interactions during clot formation [5,11]. These complementary characteristics suggest that gelatin–chitosan composite sponges represent a promising strategy for developing multifunctional hemostatic biomaterials suitable for oral surgical applications.

Although numerous gelatin–chitosan composite sponges have demonstrated promising hemostatic performance, most previous studies have primarily focused on general wound management, trauma care, or the optimization of individual material properties, such as swelling capacity, antibacterial activity, or mechanical strength [4,11,12]. Relatively few investigations have systematically examined how variations in gelatin–chitosan composition influence scaffold microstructure, physicochemical characteristics, degradation behavior, and blood–material interactions, and how these interconnected factors collectively determine hemostatic performance [5,8,11]. Consequently, the composition–structure–function relationship governing the hemostatic behavior of gelatin–chitosan composite sponges remains insufficiently understood. Furthermore, although fish-derived gelatin and chitosan have individually demonstrated favorable biological and hemostatic properties, studies integrating comprehensive physicochemical characterization, in vitro coagulation assessment, and in vivo validation in a clinically relevant oral surgical model remain limited [1,5,7,8]. Most available reports evaluate hemostatic efficacy using general wound or soft-tissue bleeding models, which may not adequately represent the unique clinical requirements of oral and maxillofacial surgery, where rapid bleeding control, scaffold stability, and favorable biodegradation are essential for successful healing [1,7]. Therefore, further investigation is required to establish how gelatin–chitosan composition regulates scaffold architecture, blood–material interactions, and hemostatic efficacy within an integrated experimental framework.

While gelatin–chitosan composite sponges have previously been investigated as hemostatic biomaterials, the relationship between polymer composition, physicochemical characteristics, and biological performance remains important for optimizing their application in specific surgical environments. Therefore, this study systematically evaluated fish-derived gelatin–chitosan sponges with different polymer ratios and integrated physicochemical characterization with in vitro biological assessment and in vivo validation. This approach was intended to elucidate how formulation-dependent changes in sponge properties relate to hemostatic performance, biocompatibility, degradation behavior, and tissue response in the context of oral and maxillofacial surgery.

## 2. Results

The physicochemical properties of gelatin–chitosan composite sponges were first characterized to evaluate the influence of gelatin-to-chitosan composition on the structural and functional properties of the materials.

### 2.1. Physicochemical Properties of Gelatin-Chitosan Sponges

#### 2.1.1. FTIR Analysis

**Figure 1:**
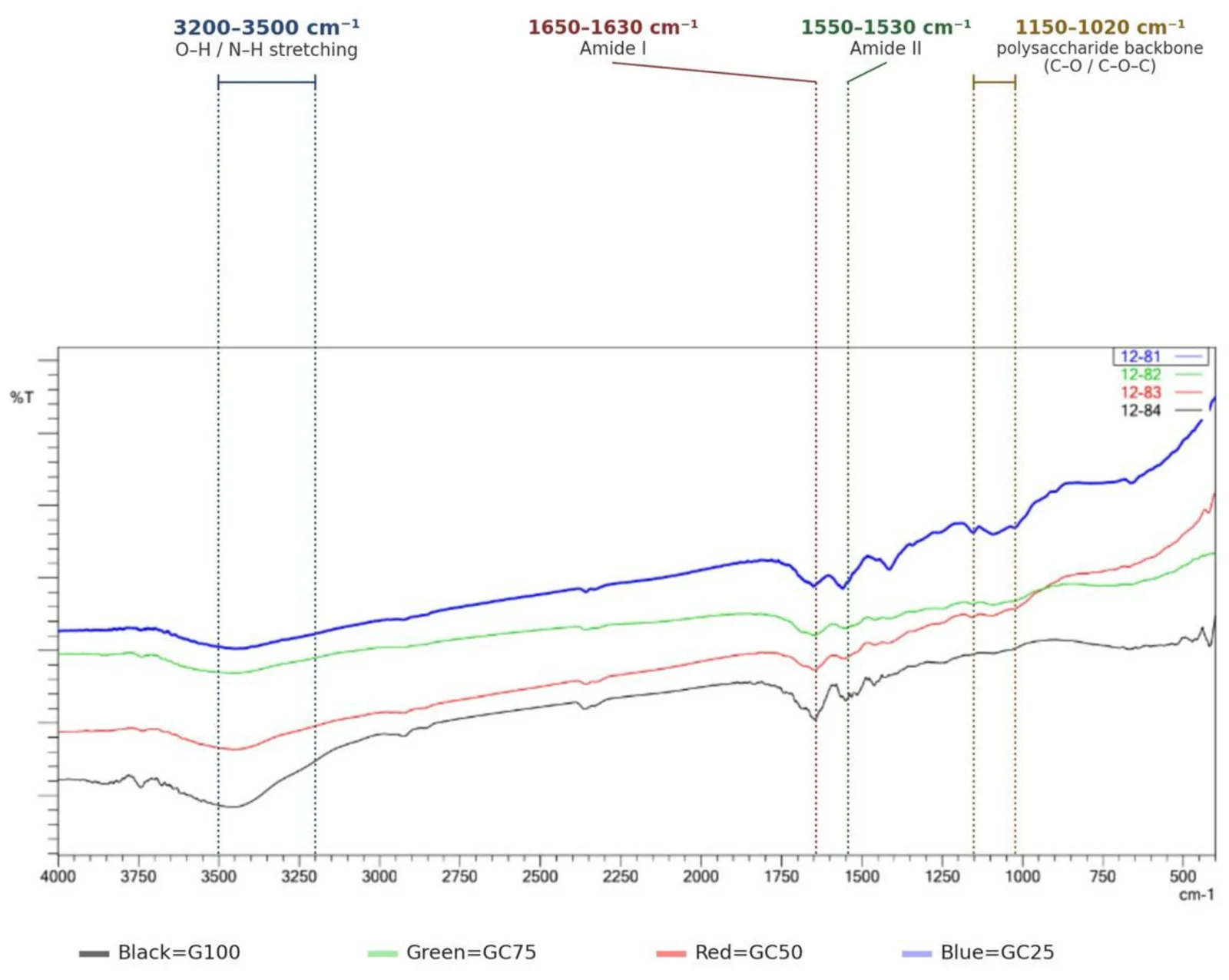
FTIR spectra of G100, GC75, GC50, and GC25 freeze-dried gelatin–chitosan sponges.

The FTIR spectra of G100, GC75, GC50, and GC25 are presented in Figure 1. All formulations exhibited comparable spectral profiles, indicating that the characteristic functional groups of gelatin and chitosan were retained after fabrication.

A broad absorption band was observed in the 3200–3500 cm^−1^ region, corresponding to overlapping O–H and N–H stretching vibrations. Characteristic gelatin absorption bands, including amide I (1650–1630 cm^−1^) and amide II (1550–1530 cm^−1^), were detected in all formulations. In addition, absorption bands within the 1150–1020 cm^−1^ region, attributed to the polysaccharide backbone of chitosan, remain evident in the composite sponges.

Differences among the formulations were primarily reflected by variations in band intensity and slight peak shifts as the gelatin-to-chitosan ratio changed, whereas no additional absorption bands were observed throughout the investigated spectral range.

#### 2.1.2. Microstructure Analysis

**Figure 2:**
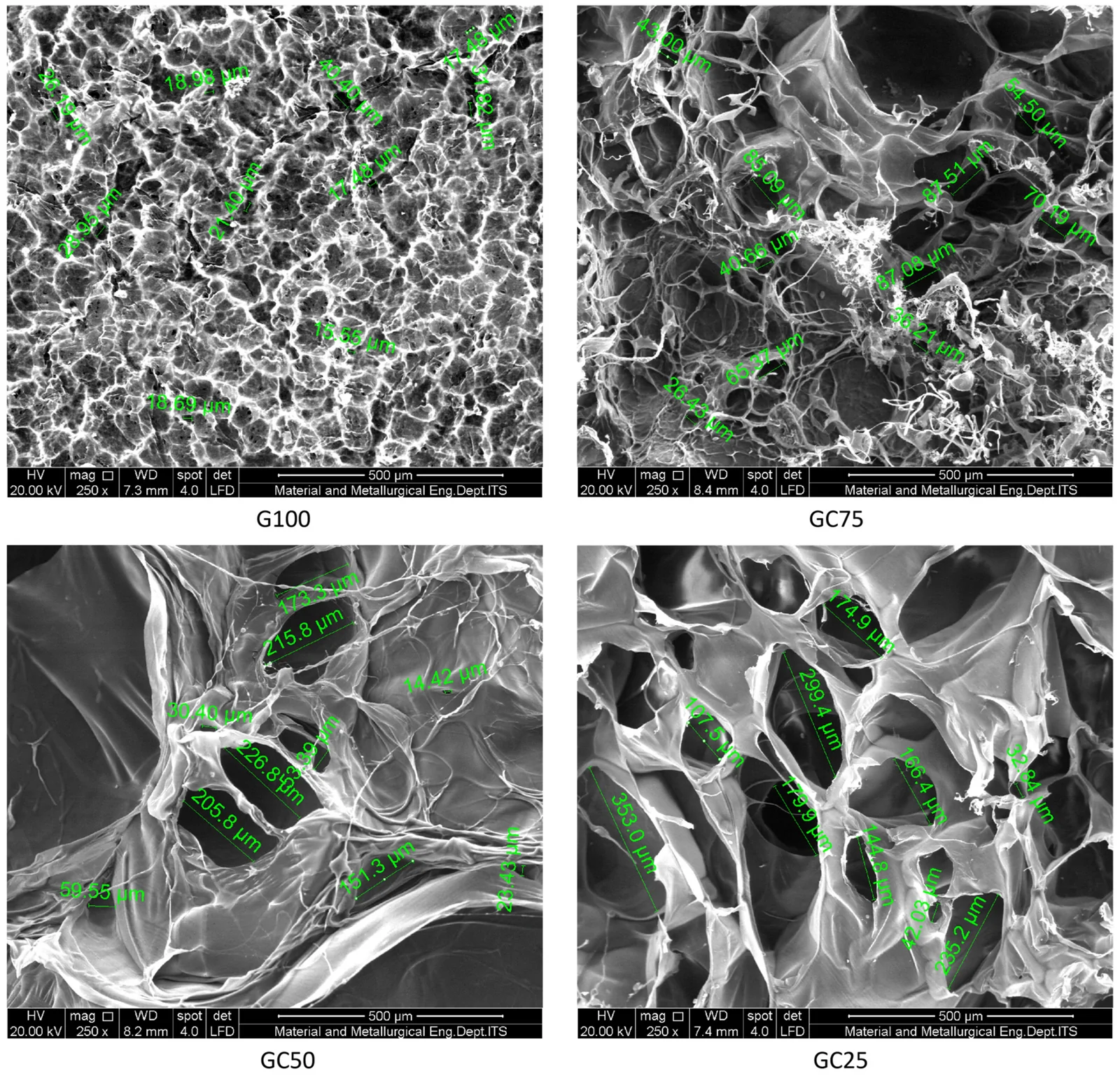
SEM micrographs of G100, GC75, GC50, and GC25.

Scanning electron microscopy (SEM) was performed to evaluate the microstructure of the freeze-dried gelatin–chitosan sponges. Representative SEM micrographs are presented in Figure 2. All formulations exhibited interconnected porous structures, although differences in pore morphology and pore distribution were observed among the formulations.

**Figure 3:**
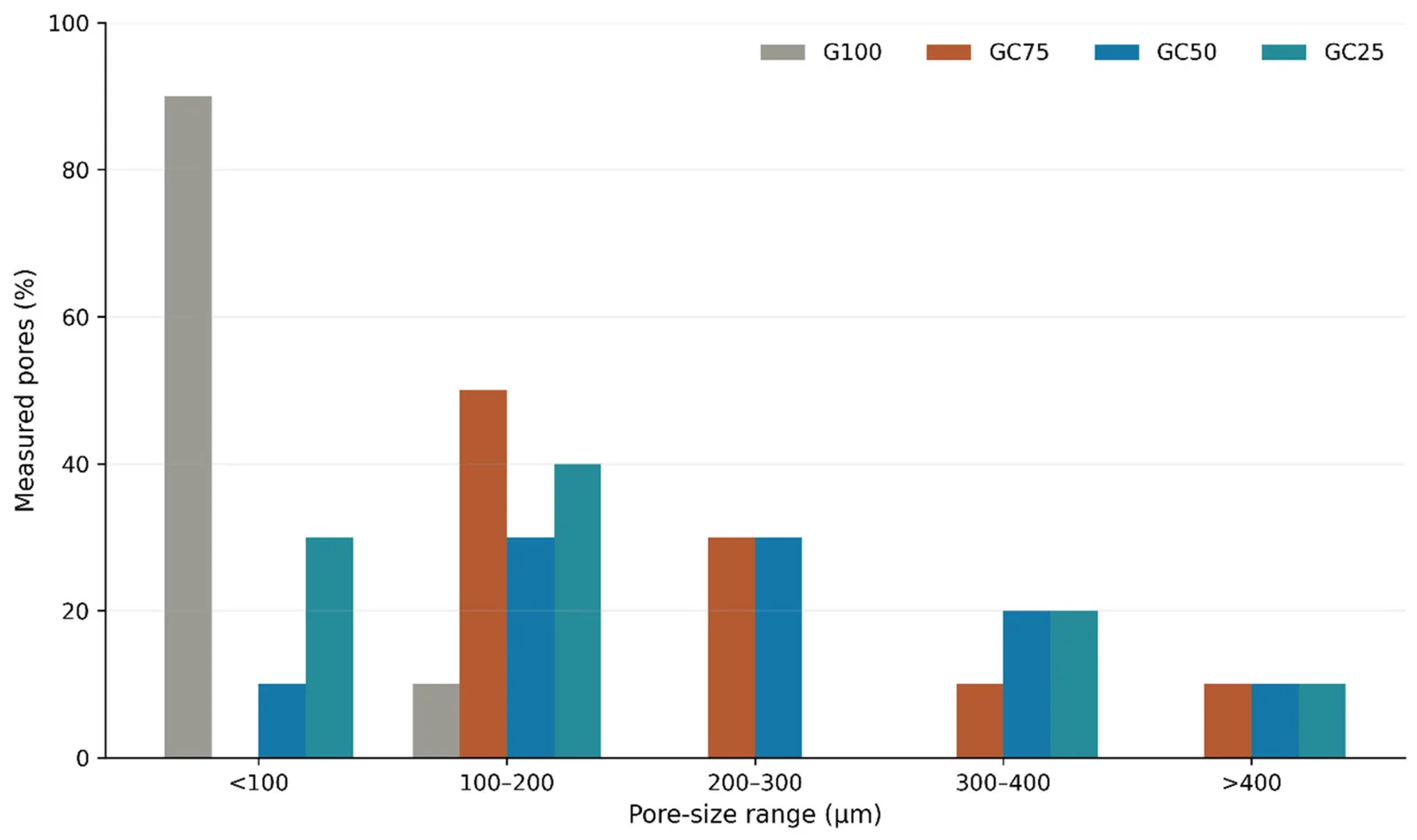
Pore-size distribution.

SEM analysis revealed composition-dependent differences in the pore architecture of the freeze-dried sponges. Quantitative ImageJ analysis showed 2D pore area fractions of 62.70%, 63.86%, 67.24%, and 56.69% for G100, GC75, GC50, and GC25, respectively (Table 1). Morphometric analysis further demonstrated marked differences in pore dimensions among the formulations. G100 exhibited predominantly small pores, with a mean pore size of 64.86 ± 28.00 µm and 90% of the measured pores below 100 µm. In contrast, incorporation of chitosan was associated with larger and more heterogeneous pore dimensions. Mean pore sizes were 220.16 ± 122.25 µm for GC75, 231.94 ± 107.31 µm for GC50, and 193.93 ± 132.14 µm for GC25. In GC75, 80% of the measured pores were distributed between 100 and 300 µm, whereas GC50 exhibited a broader distribution extending from <100 to >400 µm. GC25 also showed a heterogeneous distribution, predominantly within the <200 µm range. The pore-size distributions of all formulations are presented in Figure 3.

#### 2.1.3. Compressive Strength

**Figure 4:**
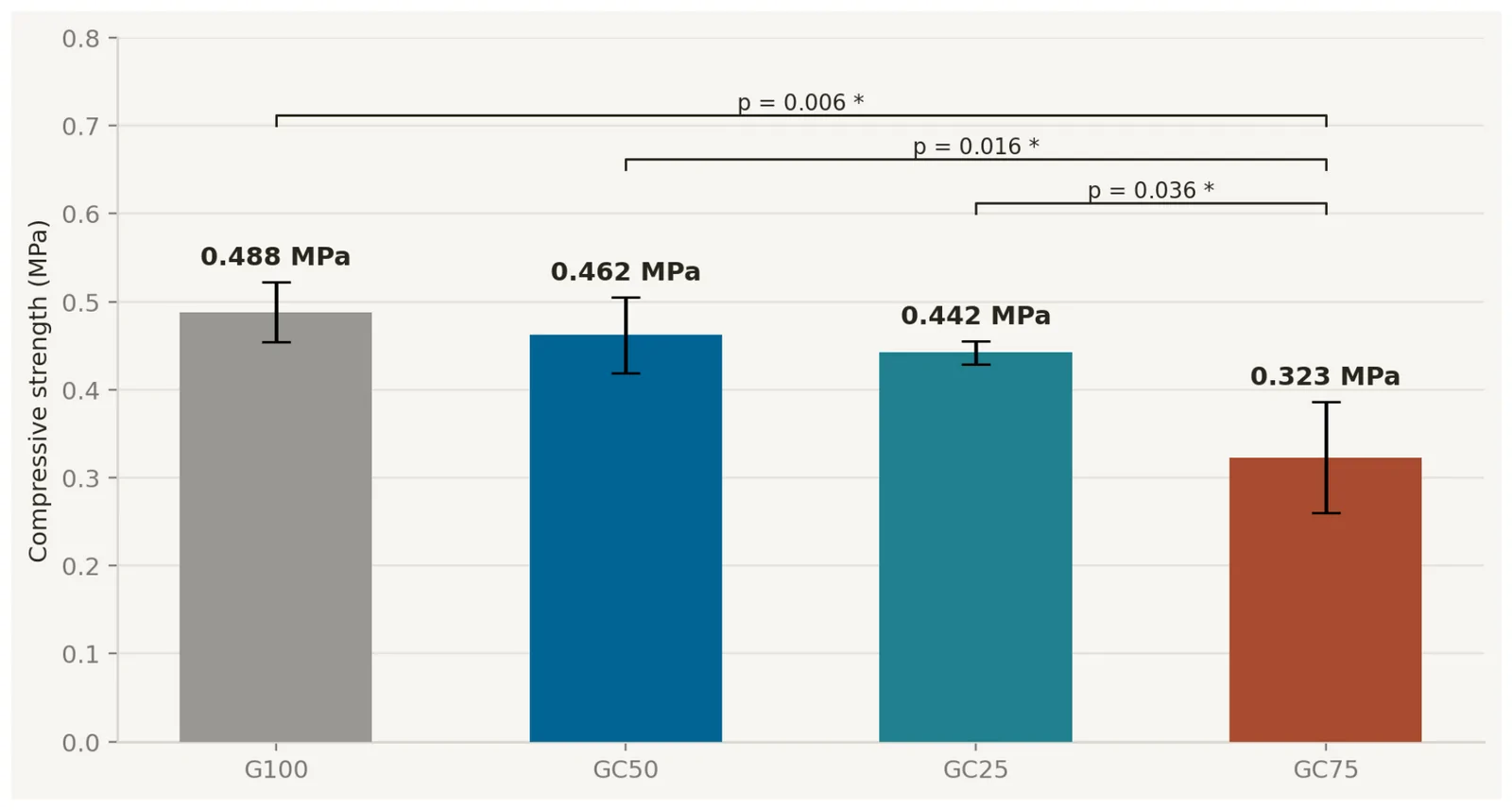
Compressive strength evaluation. * indicates a statistically significant difference between groups (*p* < 0.05).

The compressive strength varies among the gelatin–chitosan sponge formulations (Figure 4). G100 exhibited the highest compressive strength (0.488 MPa), followed by GC50 (0.462 MPa), GC25 (0.442 MPa), and GC75 (0.323 MPa). The measured compressive strength values range from 0.323 to 0.488 MPa.

#### 2.1.4. Swelling Propertiesy

The swelling properties of the gelatin–chitosan sponges were evaluated by measuring the swelling ratio after immersion in phosphate-buffered saline (PBS) for 1, 5, 10, and 30 min. The results are summarized in Table 2.

Among the composite formulations, GC75 exhibited the highest swelling ratio at 5 min (1115.22 ± 137.94%), whereas GC25 and GC50 exhibited swelling ratios of 479.51 ± 91.67% and 252.50 ± 56.18%. At 10 and 30 min, the swelling ratios of the composite sponges ranged from approximately 532% to 619%. No statistical significance among the groups (*p* < 0.0167).

#### 2.1.5. Degradation Characteristics of Gelatin-Chitosan Sponges

The in vitro degradation profiles of the gelatin–chitosan sponges following incubation in phosphate-buffered saline (PBS) at 37 °C for 7 days are summarized in Table 3. Degradation rate was measured on days 3, 5, and 7 days.

G100 exhibited complete degradation by day 3, with no remaining samples detected throughout the subsequent observation period. In contrast, all composite formulations remained intact throughout the 7-days incubation period.

Among the composite sponges, GC25 has the lowest degradation rate after 7 days of PBS immersion (15.81 ± 2.76 mg), followed by GC50 (20.64 ± 5.68 mg) and GC75 (48.73 ± 5.07 mg). These findings indicate that GC25 exhibited the slowest degradation profile throughout the observation period, whereas GC75 underwent the largest mass loss among the composite formulations.

### 2.2. BiologicalProperties

Based on the physicochemical characterization, GC75 and GC25 were selected for further biological assessment of cytocompatibility and antibacterial activity.

#### 2.2.1. Cell Viability

The cell viability of the selected gelatin–chitosan composite sponges were evaluated using the MTT assay after 24 h of incubation with human gingival fibroblasts. The negative control exhibited the highest cell viability (100.00 ± 20.34%), followed by GC25 (85.13 ± 16.64%), the positive control (82.92 ± 15.59%), and GC75 (77.10 ± 10.32%). One-way ANOVA revealed a significant difference in cell viability among the experimental groups. Post hoc Tukey’s multiple comparison test showed a significant difference only between GC75 and the negative control, whereas no significant differences were observed between GC25 and either the negative or positive control groups. All tested sponge formulations exhibited cell viability values above 70%.

#### 2.2.2. Antibacterial Activity

The antibacterial activity of the selected gelatin–chitosan composite sponges were evaluated using the agar diffusion method. No inhibition zone was detected around GC75, GC25, or the commercial control (Curaspon^®^), indicating that the tested materials did not exhibit observable antibacterial activity under the experimental conditions. Bacterial growth extended to the edges of all samples, indicating the absence of detectable growth inhibition under the experimental conditions. Accordingly, no antibacterial activity was observed for either composite formulation or the commercial control in the agar diffusion assay.

### 2.3. In Vitro Hemostatic Evaluation

#### 2.3.1. BCI Analysis

Table 4 presents BCI values of the tested hemostatic sponges, including the gelatin–chitosan composite formulations (GC75 and GC25) and the commercial gelatin sponge (Curaspon^®^). The BCI assay evaluates the clotting ability of a material by measuring the absorbance of hemoglobin released from non-coagulated red blood cells.

As shown in Table 4, both gelatin–chitosan composite sponges exhibited lower BCI values compared with the commercial gelatin sponge. The GC75 formulation showed the lowest BCI value (91.42 ± 4.06), followed by GC25 (92.54 ± 4.95), whereas the commercial sponge (Curaspon^®^) exhibited the highest value (100.97 ± 1.75). Lower BCI values indicate more effective clot formation, suggesting that the composite sponges enhanced blood coagulation compared with the commercial control.

Statistical analysis demonstrated a significant difference among the tested groups (*p* < 0.001). Among the tested formulations, the GC75 sponge showed the most favorable clotting performance in the BCI assay.

#### 2.3.2. BCT Analysis

**Figure 5:**
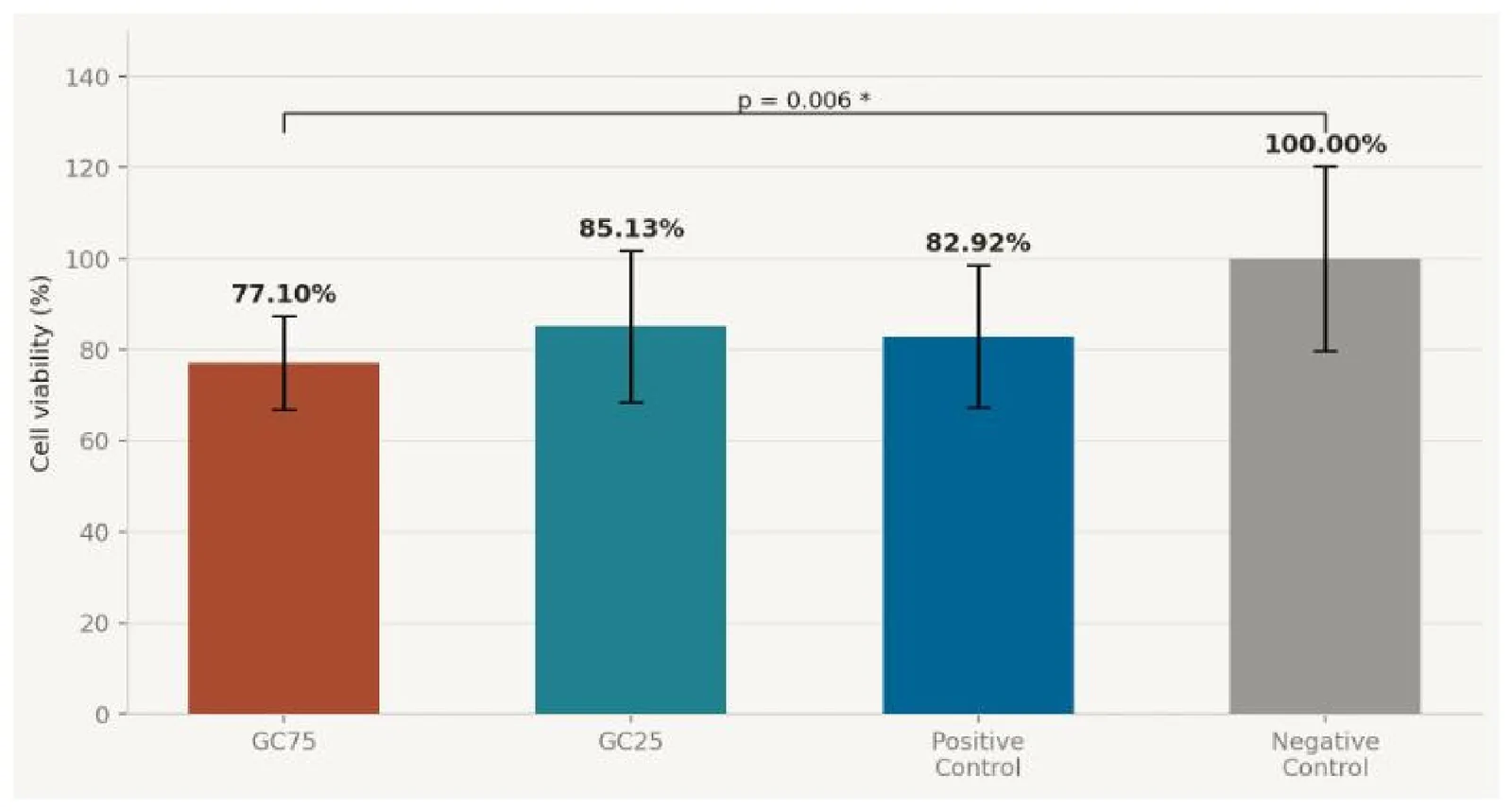
Cell viability evaluation. Data are presented as mean ± SD. * indicates a statistically significant difference between groups (*p* < 0.05).

The BCT assay was performed to evaluate the ability of the hemostatic materials to accelerate whole-blood coagulation. The results of the BCT analysis are summarized in Table 5, and the clotting time distribution is illustrated in the Kaplan–Meier survival curves (Figure 5).

The GC75 composite sponge exhibited the shortest clotting time, with a mean value of 8.61 ± 0.29 min. The commercial gelatin sponge (Curaspon^®^) showed a mean clotting time of 9.87 ± 0.44 min, whereas the GC25 formulation required a longer clotting time (11.99 ± 2.07 min). The control group, consisting of blood without sponge material, exhibited the longest clotting time (13.87 ± 0.60 min), indicating slower spontaneous coagulation.

Kaplan–Meier survival analysis revealed a statistically significant difference among the tested groups (log-rank test, *p* = 0.001). As shown in Figure 2, the GC75 group achieved complete clot formation earlier than the other groups, with all samples forming stable clots at approximately 8.8 min.

#### 2.3.3. Fibrin Generation

**Figure 6:**
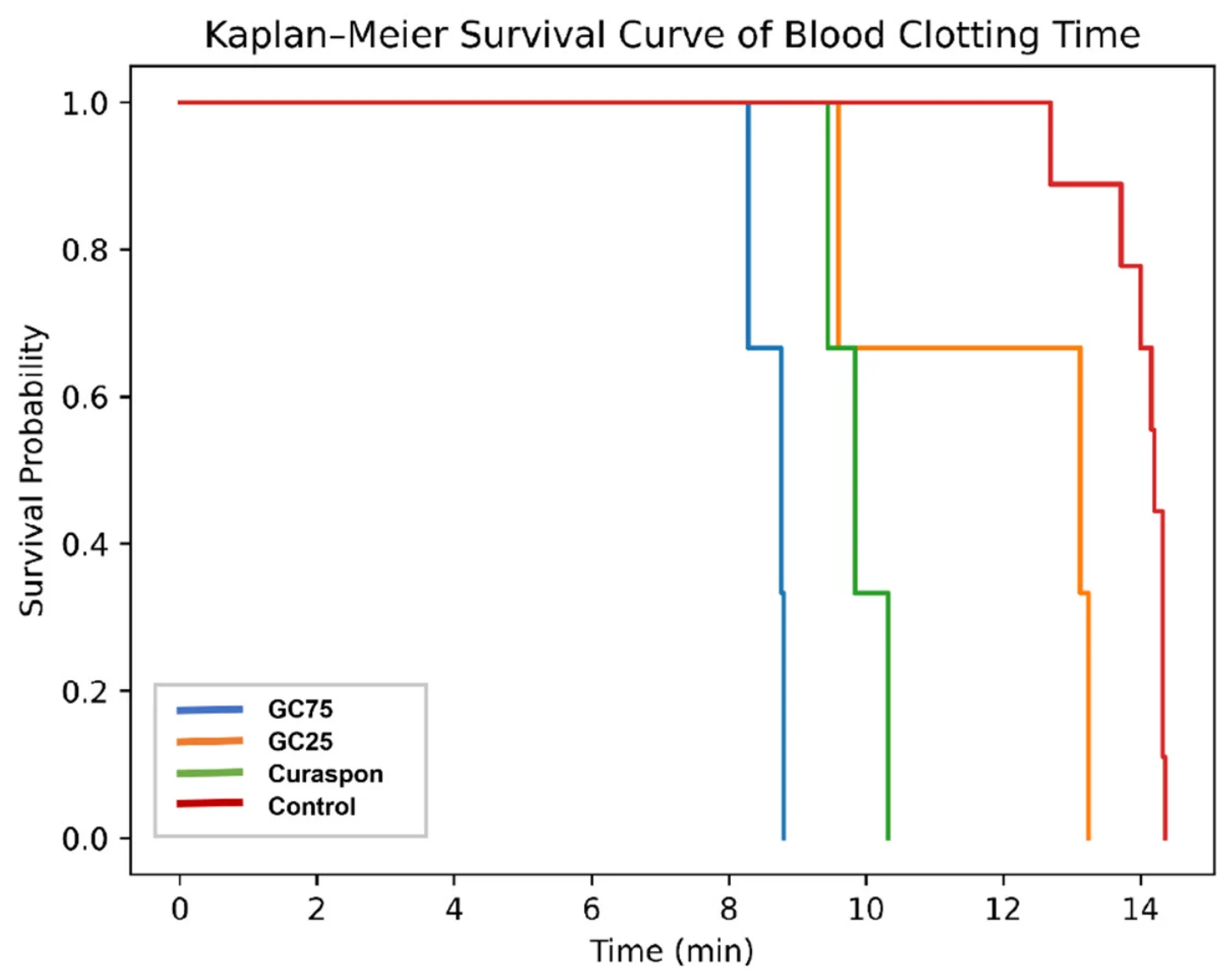
Kaplan–Meier survival curves showing the BCT distribution for GC75, GC25, Curaspon^®^, and control samples.

The fibrin generation assay was performed to evaluate fibrin formation during the coagulation process using turbidity measurements. The results demonstrated that the GC75 composite sponge exhibited the highest fibrin generation activity compared with the GC25 formulation, the commercial sponge (Curaspon^®^), and the control group. This finding indicates that the GC75 formulation was more effective in promoting fibrin polymerization during the coagulation process (Figure 6).

Statistical analysis using the Kruskal–Wallis test showed a significant difference among the tested groups (*p* < 0.005). Further pairwise comparisons using Dunn’s post hoc test revealed significant differences between the GC75 group and the commercial sponge (*p* < 0.05), as well as between the GC75 group and the control group (*p* < 0.002).

#### 2.3.4. PT

**Figure 7:**
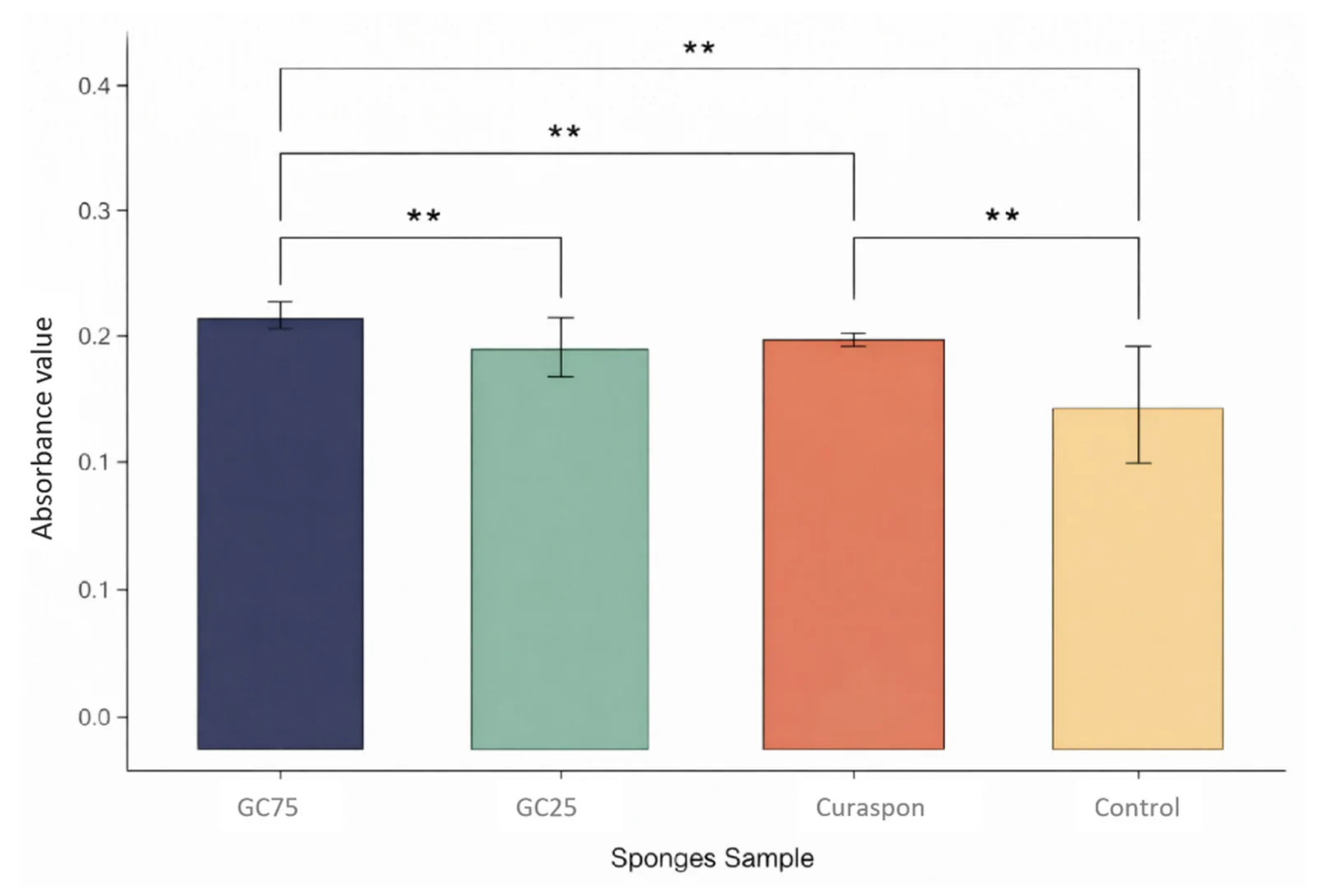
Fibrin generation assay comparing fibrin formation among gelatin–chitosan sponges (GC75 and GC25), commercial sponge (Curaspon^®^), and control. ** indicates a statistically significant difference (*p* < 0.01).

**Figure 8:**
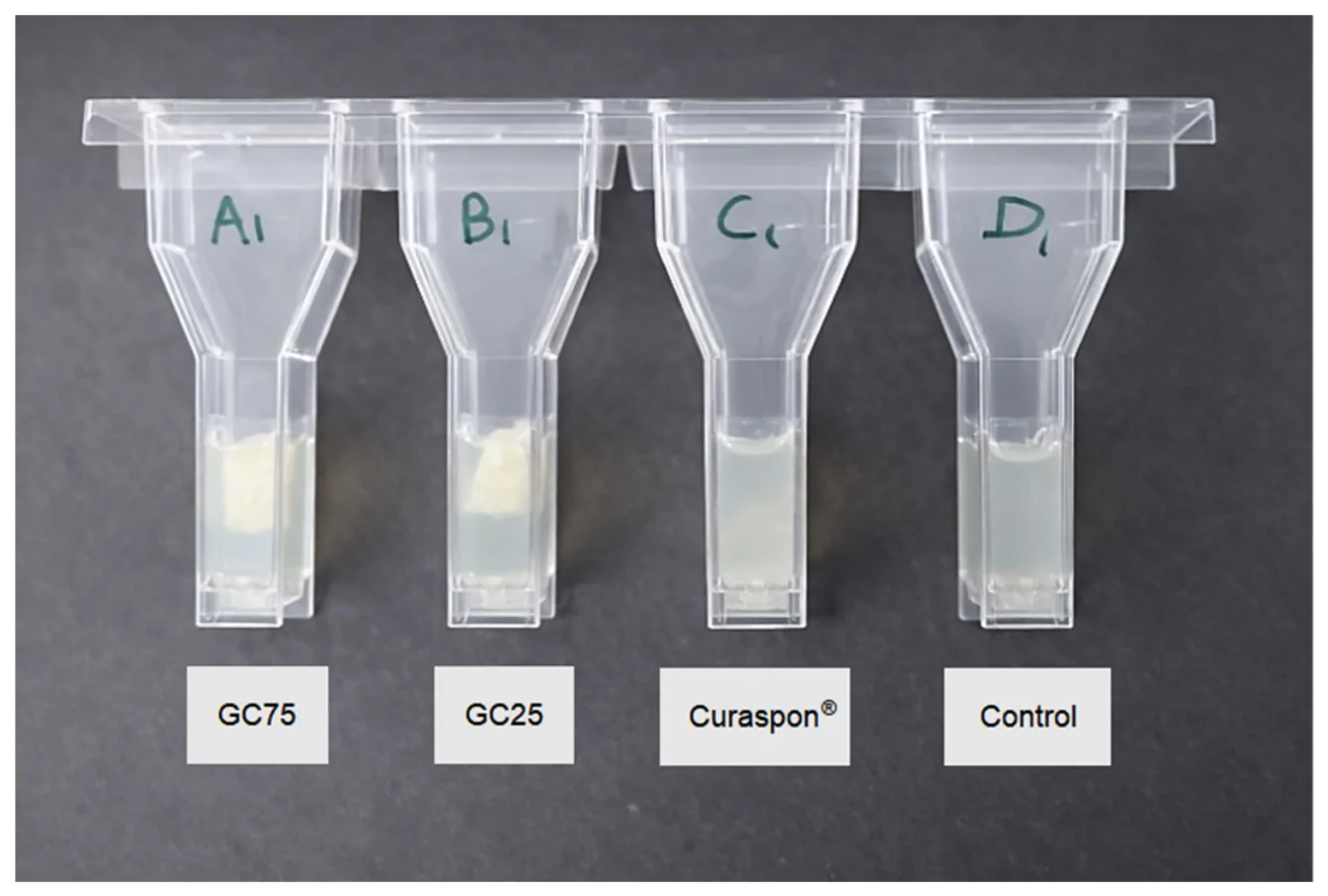
Experimental setup for PT measurement using a four-segment cuvette. The labels A1, B1, C1, and D1 are temporary sample identifiers used during the experimental procedure and do not represent experimental variables or analytical measurements.

PT was measured to evaluate the activation of the extrinsic coagulation pathway. The experimental setup used for PT measurement is shown in Figure 7, and the obtained PT values are summarized in Figure 8.

The results showed that the GC75 composite sponge exhibited a mean PT value of 12.8 ± 2.52 s, which was slightly lower than those observed for the other sponge samples. However, the PT values of all tested materials remained within the normal reference range of the test kit (10–15 s).

Statistical analysis indicated that a significant difference was observed only between the GC25 formulation and the control group. Overall, the PT results suggest that the gelatin–chitosan composite sponges did not markedly alter the extrinsic coagulation pathway compared with the commercial sponge.

#### 2.3.5. PTT

**Figure 9:**
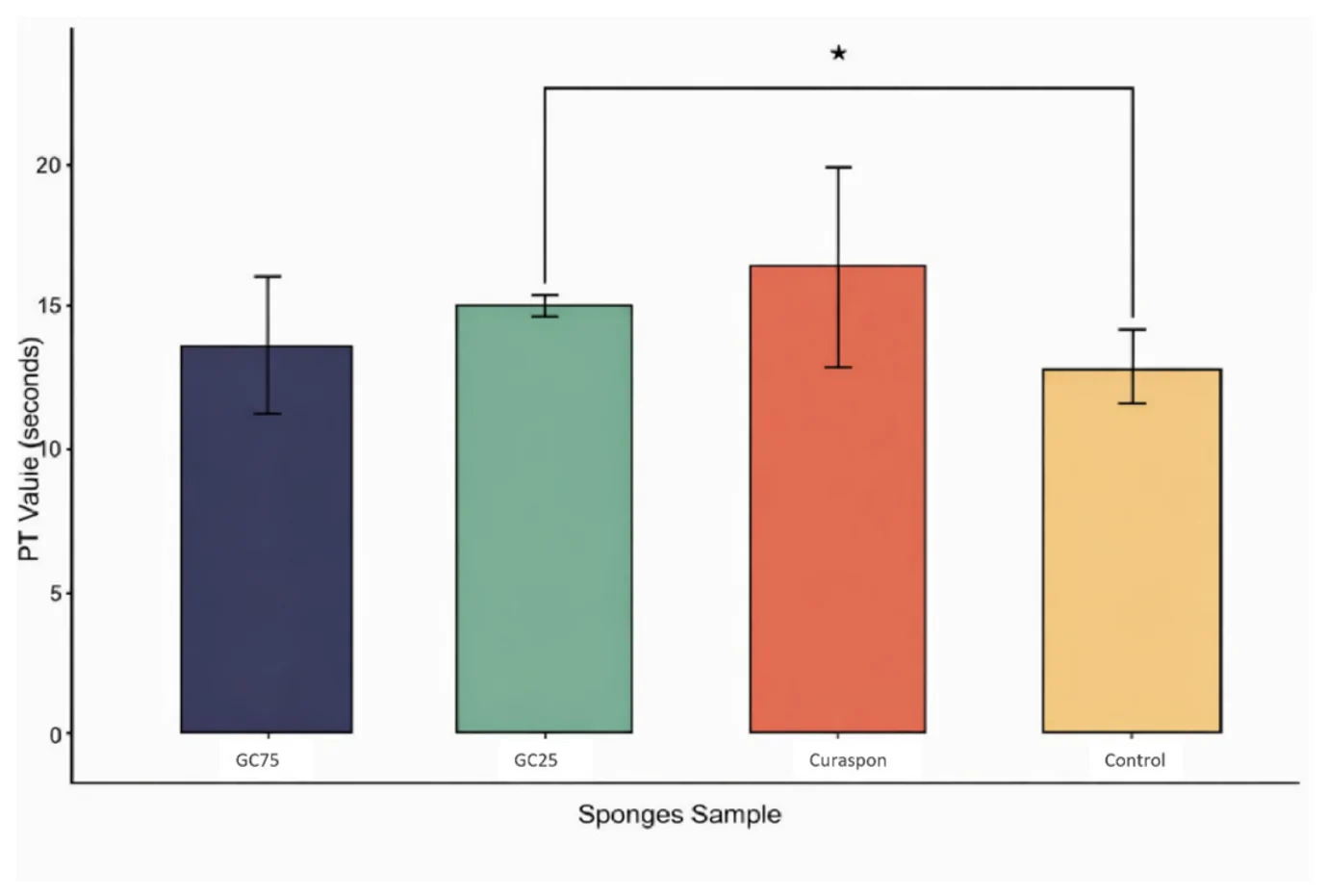
Comparison of prothrombin time (PT) among gelatin–chitosan sponges (GC75 and GC25), commercial sponge (Curaspon^®^), and control. Data are presented as mean ± SD. * *p* < 0.05 indicates a statistically significant difference between the indicated groups.

PTT was evaluated to assess the intrinsic coagulation pathway. The measured PTT values for the tested materials are presented in Figure 9.

The commercial sponge (Curaspon^®^) showed the lowest mean PTT value (28.3 s), followed by the GC25 formulation (29.73 s) and the GC75 formulation (32.43 s). Despite these numerical differences, statistical analysis revealed no significant differences among the tested groups.

These findings indicate that the gelatin–chitosan composite sponges produced coagulation responses comparable to the commercial hemostatic sponge in the PTT assay.

Overall, GC75 consistently demonstrated superior hemostatic performance across multiple coagulation assays compared with GC25 and the commercial control. A consolidated summary of the in vitro hemostatic evaluation is presented in Table 6.

### 2.4. In Vivo Hemostatic Performance

#### 2.4.1. Bleeding Time

Bleeding time was evaluated following tooth extraction to assess the in vivo hemostatic performance of the composite sponge. The descriptive statistics of bleeding time for each experimental group on postoperative days 1, 3, and 7 are presented in Table 7.

Two-way ANOVA demonstrated a significant effect of treatment group on bleeding time, whereas neither observation day nor the interaction between treatment group and observation day showed a statistically significant effect. Post hoc Bonferroni analysis revealed significantly shorter bleeding times in the GC75 group than in the negative control group, while no significant difference was observed between the GC75 and positive control groups.

#### 2.4.2. PMN Infiltration

Polymorphonuclear neutrophil (PMN) infiltration was evaluated to assess the inflammatory response following tooth extraction. Significant differences in PMN infiltration among the experimental groups were observed on postoperative days 1 (*p* = 0.015) and 7 (*p* = 0.012), whereas no significant difference was detected on postoperative day 3 (*p* = 0.271). On postoperative day 1, post hoc analysis revealed a significant difference between the negative control and GC75 groups. Significant difference was observed postoperative day 7 between the positive control and GC75 groups. The GC75 group exhibited higher median PMN infiltration scores than the other experimental groups on postoperative days 1 and 7.

#### 2.4.3. Biomaterial Degradation

In vivo biomaterial degradation was evaluated by histopathological examination on postoperative days 1, 3, and 7. Biomaterial degradation was observed in three of four samples (75.0%) in the GC75 group on postoperative days 1 and 7. On postoperative day 3, degradation was detected in one of four samples (25.0%) in both the positive control and GC75 groups. Fisher–Freeman–Halton exact test indicated that the differences in the proportion of biomaterial degradation among the experimental groups were not statistically significant on postoperative days 1, 3, or 7.

#### 2.4.4. Hematological and Blood Biochemical Analyses

Hematological and blood biochemical analyzes were performed to evaluate the systemic response following biomaterial implantation. The measured parameters included leukocyte, neutrophil, lymphocyte, monocyte, aspartate aminotransferase (AST), alanine aminotransferase (ALT), urea, and creatinine. Hematological and biochemical values varied according to the experimental group and observation period. Two-way analysis of variance (ANOVA) revealed significant effects of treatment group, observation day, or their interaction for several parameters. Likewise, the Kruskal–Wallis test identified significant differences among groups at specific observation time points. However, subsequent pairwise comparisons using the Mann–Whitney U test with Bonferroni correction did not identify any statistically significant differences between individual groups. The GC75 group did not exhibit consistent statistically significant differences compared with either the negative control or the positive control group with respect to leukocyte, neutrophil, lymphocyte, monocyte, AST, ALT, urea, or creatinine levels.

## 3. Discussion

### 3.1. Effect of Gelatin–Chitosan Composition on Physicochemical Properties

Gelatin provides a highly hydrophilic protein matrix that facilitates rapid fluid absorption and supports cell–material interactions, whereas chitosan contributes structural reinforcement through its polycationic molecular backbone and extensive hydrogen-bonding capability [8,12]. Composite sponge of gelatin and chitosan has dual action hemostatic mechanism, gelatin as structural matrix, can absorb liquid 40 times its own weight, while positively charged chitosan bonds with negative charged RBC to reach hemostatic [3]. Consequently, balancing these two polymers is essential for producing a scaffold capable of absorbing blood rapidly while maintaining sufficient structural integrity during the early phase of hemostasis. Similar composition-dependent behavior has also been demonstrated in gelatin-based macro-porous sponges developed for wound healing applications [5].

The FTIR analysis further supports this interpretation by demonstrating that all formulations exhibited the characteristic absorption bands of gelatin and chitosan without the appearance of additional peaks indicative of new covalent bond formation. Comparable FTIR profiles have been reported in gelatin–chitosan composite sponges fabricated without chemical crosslinkers, where intermolecular interactions dominate scaffold stabilization [5,13]. This stabilization mechanism has been recognized as a key strategy for improving the performance of naturally derived absorbable hemostatic biomaterials without compromising their biodegradability [3].

The molecular interactions identified by FTIR were subsequently reflected in the porous architecture of the freeze-dried scaffolds. Freeze-drying generates a three-dimensional interconnected pore network through ice-crystal templating, and the stability of the polymer matrix largely determines whether this architecture can be preserved after sublimation [3,12].

Quantitative pore analysis further demonstrated that incorporation of chitosan substantially altered the pore architecture of the freeze-dried scaffolds. Whereas G100 was dominated by pores below 100 µm, the composite formulations exhibited larger and more heterogeneous pore dimensions. GC75 showed a mean pore size of approximately 220 µm, with most measured pores distributed between 100 and 300 µm. Interestingly, GC50 exhibited both a slightly higher 2D pore area fraction and mean pore size than GC75, indicating that pore size or pore area alone does not determine hemostatic performance. Rather, the functional behavior of the scaffold likely reflects the combined effects of pore architecture, fluid absorption, polymer composition, mechanical stability, and degradation behavior [14].

The observed differences in pore architecture provide a structural explanation for the swelling behavior of the composite scaffolds. Highly interconnected pores facilitate rapid penetration and distribution of aqueous fluids through capillary action, whereas continuous pore walls help preserve scaffold integrity during liquid absorption [8,12].

Although SEM-based morphometric analysis enabled quantitative evaluation of pore size, pore-size distribution, and two-dimensional pore-area fraction, three-dimensional pore volume and specific surface area were not directly determined in the present study. These parameters require dedicated three-dimensional or surface-characterization techniques and should be investigated in future studies.

In addition, the surface zeta potential of the fabricated sponges was not directly measured. Therefore, the proposed electrostatic interaction between chitosan amino groups and negatively charged blood components should be regarded as a literature-supported mechanistic interpretation rather than direct experimental evidence from the present study.

In the present study, all composite formulations showed high swelling capacity, although the magnitude and time swelling profiles differed between formulations, with GC75 exhibiting the highest mean swelling ratio while maintaining structural stability the immersion period. These findings suggest that the pore architecture of GC75 provided an appropriate balance between water uptake and mechanical preservation, which is considered a desirable characteristic for absorbable hemostatic biomaterials [3,13].

In vitro degradation results supported the contribution of chitosan to scaffold stability under physiological conditions. An ideal biodegradable hemostatic sponge should remain structurally intact during the initial stage of hemostasis before gradually degrading as tissue repair progresses [8]. Excessively rapid degradation may compromise clot stabilization because the scaffold loses its physical support before fibrin maturation is complete, while prolonged persistence may delay tissue remodeling. Similar degradation characteristics have been described in gelatin–chitosan composite sponges stabilized predominantly through hydrogen bonding and polymer chain involvement [5,13].

The compressive strength results should be interpreted together with the swelling and degradation data rather than as an isolated mechanical parameter. Mechanical performance in porous biomaterials is closely associated with pore geometry, pore-wall thickness, and overall structural continuity rather than polymer composition alone [8,12]. GC75 exhibited the lowest compressive strength among the tested formulations (0.323 MPa), indicating that its mechanical resistance was lower than that of G100, GC50, and GC25. Therefore, the mechanical performance of GC75 should be regarded as a limitation rather than a particular advantage of this formulation. Nevertheless, for a topical absorbable hemostatic sponge, compressive strength represents only one component of functional performance and should be considered together with pore architecture, fluid absorption, and degradation behavior. The selection of GC75 for subsequent biological evaluation was therefore based on its overall physicochemical profile rather than on mechanical strength alone [5]. Consequently, achieving an optimal balance between absorbency and mechanical stability is more important than maximizing compressive strength alone for topical hemostatic applications [13].

The favorable balance achieved by GC75 indicates that the selected gelatin–chitosan ratio successfully optimized the structural organization of the freeze-dried scaffold. This integrated structure–property relationship provides a logical explanation for selecting GC75 for subsequent biological evaluation and highlights the importance of polymer composition in the rational design of naturally derived hemostatic biomaterials [3,5].

### 3.2. Biological Performance of the Gelatin–Chitosan Sponge

The favorable physicochemical characteristics identified during scaffold characterization provided the basis for subsequent biological evaluation. Among the four formulations investigated (G100, GC75, GC50, and GC25), GC75 was selected as the principal candidate for further biological assessment because it demonstrated the most balanced combination of interconnected pore architecture, swelling capacity, structural stability, and degradation behavior, whereas GC25 was included to evaluate the influence of a higher chitosan content on cellular responses. This stepwise evaluation is consistent with the development strategy of absorbable hemostatic biomaterials, in which optimization of physicochemical properties is followed by biological validation to ensure that the selected formulation remains compatible with living tissues before proceeding to functional hemostatic and in vivo evaluations [3,10].

The MTT assay demonstrated that both GC75 and GC25 maintained cell viability above the minimum acceptance threshold specified in ISO 10993-5 [15], indicating that neither formulation exhibited cytotoxic effects toward human gingival fibroblasts. Although GC75 showed significantly lower cell viability than the untreated negative control, no significant difference was observed between GC25 and either the negative control or the commercial reference material. The viability values obtained for both composite formulations remained above 70%, indicating compliance with the internationally accepted threshold for non-cytotoxic biomaterials intended for medical applications. The preservation of cellular metabolic activity observed in this study is consistent with previous reports demonstrating that gelatin–chitosan composite scaffolds provide a biologically favorable microenvironment that supports fibroblast survival and proliferation while maintaining adequate biodegradability [5,16,17].

Although gelatin and chitosan are generally considered biocompatible materials, the cytocompatibility of chitosan-containing biomaterials may also be influenced by intrinsic material characteristics, including the degree of deacetylation. The chitosan used in the present study had a supplier-reported degree of deacetylation of >85%. A higher degree of deacetylation increases the proportion of free amino groups and may consequently influence cellular interactions. Caprifico et al. (2021) [18] reported that very highly deacetylated chitosan (>90%) may exhibit slight cytotoxicity and reduced biodegradability. Therefore, the degree of deacetylation may have contributed, among other physicochemical factors, to the lower mean cell viability observed for GC75 (77.10 ± 10.32%). However, because the exact degree of deacetylation was not experimentally determined in the present study, a direct relationship between the degree of deacetylation and the observed cell viability cannot be established.

Scaffold architecture may have further contributed to the favorable biological response. Highly interconnected pores facilitate the diffusion of oxygen, nutrients, and metabolic waste, thereby creating a microenvironment that supports cell attachment and proliferation after implantation [12,16]. In addition, the balanced swelling behavior and controlled degradation profile observed for GC75 are expected to maintain temporary structural support during the early stages of tissue repair while permitting gradual scaffold remodeling. Synchronization between scaffold degradation and tissue regeneration has been recognized as a desirable characteristic of naturally derived biomaterials for regenerative medicine and hemostatic applications [3,8].

The agar diffusion assay demonstrated that neither GC75 nor GC25 produced a detectable inhibition zone against *Staphylococcus aureus*, indicating that no measurable antibacterial activity was observed under the experimental conditions employed. Similar findings were also obtained for the commercial gelatin sponge, suggesting that the absence of bacterial inhibition was not unique to the developed composite scaffold but was consistent with the biological characteristics of conventional absorbable gelatin-based hemostatic materials.

Although chitosan is widely recognized for its antibacterial properties, its activity is strongly influenced by several factors, including molecular weight, degree of deacetylation, concentration, pH, solubility, and the protonation state of its amino groups. In the present study, the antibacterial effect of chitosan was not detectable by the agar diffusion assay, as no inhibition zone was observed around either gelatin–chitosan formulation. This finding may be explained by the incorporation of chitosan within the solid gelatin–chitosan sponge matrix, which may restrict its release and diffusion into the surrounding agar. In addition, the neutralization and washing steps used during sponge preparation may reduce the protonation of chitosan amino groups. Because the antibacterial activity of chitosan is largely associated with positively charged protonated amino groups interacting with negatively charged bacterial cell surfaces, reduced protonation under near-neutral conditions may further limit its detectable antibacterial effect. Therefore, the absence of an inhibition zone in this study should be interpreted as the absence of detectable antibacterial activity under the specific agar diffusion conditions employed, rather than as evidence that chitosan inherently lacks antibacterial properties [11,13].

Another important consideration is the limitation of the agar diffusion method itself. This assay primarily evaluates the diffusion of antimicrobial compounds from the biomaterial into the surrounding agar rather than direct contact-mediated antibacterial activity. Recent reviews have emphasized that chitosan incorporated into crosslinked or composite scaffolds may remain immobilized within the polymer network, thereby restricting molecular diffusion despite retaining surface-associated biological activity [19]. Consequently, composite biomaterials may demonstrate negligible inhibition zones while still maintaining satisfactory biological performance during tissue repair [19].

Following biological safety evaluation, the functional capacity of the scaffold to promote coagulation was investigated using complementary in vitro hemostatic assays, including the BCI, BCT, fibrin generation, PT, and activated PTT. These assays evaluate different aspects of coagulation and collectively provide a comprehensive assessment of the interaction between biomaterials and blood components rather than relying on a single parameter [3,20,21].

Among the evaluated formulations, GC75 consistently demonstrated the most favorable hemostatic profile. The BCI assay showed that GC75 exhibited the lowest BCI value among all materials, indicating a more efficient mesh of erythrocytes within the developing clot than GC25 and the commercial gelatin sponge. Because lower BCI values reflect more rapid clot formation, these findings indicate that the incorporation of chitosan into the gelatin matrix enhanced the ability of the scaffold to concentrate blood components during the initial phase of coagulation [3,12]. Similar reductions in BCI have been reported in porous gelatin–chitosan sponges with highly interconnected pore structures that facilitate rapid blood absorption and clot stabilization [5,20].

The blood clotting time assay further supported these observations. GC75 achieved complete clot formation earlier than GC25, the commercial gelatin sponge, and the untreated control, indicating a greater capacity to accelerate whole-blood coagulation. This behavior is closely associated with the physicochemical properties discussed previously. The interconnected porous architecture and high swelling capacity of GC75 promote rapid capillary-driven blood absorption, leading to localized enrichment of erythrocytes, platelets, and coagulation factors within the scaffold matrix [3,12]. Such concentration effects facilitate platelet activation and accelerate fibrin formation, thereby shortening the overall clotting time [20,21].

The fibrin generation assay provided additional evidence supporting the superior biological performance of GC75. The enhanced fibrin generation observed for this formulation indicates more efficient fibrin polymerization and stabilization of the developing thrombus than in the other tested groups. Fibrin formation represents the final common pathway of blood coagulation and plays a central role in stabilizing platelet aggregates and entrapped erythrocytes within the clot [3,20]. Therefore, the higher fibrin generation exhibited by GC75 is consistent with the improvements observed in both the BCI and BCT assays, collectively suggesting a more efficient local hemostatic response.

Interestingly, despite the improvements observed in BCI, BCT, and fibrin generation, PT and PTT values remained comparable among the tested formulations and the commercial control. This finding is biologically plausible because PT and PTT primarily evaluate the integrity of the extrinsic and intrinsic coagulation pathways under standardized plasma conditions rather than localized interactions occurring at the biomaterial surface [3,21]. Consequently, enhancement of localized clot formation does not necessarily require measurable alterations in systemic coagulation parameters. Such behavior is considered advantageous for topical hemostatic biomaterials because effective bleeding control can be achieved without directly disturbing physiological coagulation pathways [4,20].

The superior hemostatic performance of GC75 can therefore be explained by the synergistic contribution of its physicochemical and biological characteristics. GC75 exhibited a favorable balance of pore architecture, swelling capacity, and controlled degradation. These structural characteristics facilitate rapid blood infiltration and retention within the scaffold, while gelatin provides a hydrophilic matrix that readily absorbs plasma and chitosan promotes electrostatic interactions with erythrocytes and platelets through its positively charged amino groups [5,11]. The combined effects of rapid fluid absorption, localized concentration of coagulation factors, platelet activation, and accelerated fibrin polymerization provide a mechanistic explanation for the superior performance of GC75 across multiple in vitro hemostatic assays [20,21].

The biological evaluation demonstrates that GC75 successfully integrates acceptable cytocompatibility with efficient coagulation performance while maintaining biological behavior comparable to commercially available absorbable gelatin sponges. Although intrinsic antibacterial activity was not detected under the agar diffusion conditions employed, the composite scaffold fulfilled its primary design objective as a biodegradable topical hemostatic material. These in vitro findings provide a strong rationale for the subsequent in vivo evaluation, in which the hemostatic efficacy and host tissue response of GC75 are further validated under physiological conditions.

In vitro biological evaluation demonstrated that the gelatin–chitosan composite scaffold achieved a favorable balance between biological compatibility and hemostatic functionality. The incorporation of chitosan into the gelatin matrix did not compromise fibroblast viability, while the optimized formulation (GC75) consistently exhibited superior coagulation performance through lower blood clotting index values, shorter blood clotting time, and enhanced fibrin generation. These findings indicate that optimization of the gelatin-to-chitosan ratio successfully improved local hemostatic performance without adversely affecting cellular compatibility or altering the physiological coagulation pathways represented by PT and PTT [3,5,20].

The biological performance observed in the present study should be interpreted as the result of synergistic interactions among the physicochemical characteristics discussed in the previous section. Rather than acting independently, pore architecture, swelling behavior, degradation kinetics, and molecular interactions collectively regulate blood infiltration, concentration of coagulation components, platelet activation, and stabilization of the developing fibrin network [3,12]. Recent reviews have emphasized that the efficacy of modern absorbable hemostatic biomaterials depends on this integrated structure–function relationship rather than on any single material property [4,21].

Although the in vitro findings provide strong evidence supporting the biological potential of GC75, these experimental models cannot fully reproduce the complex physiological environment encountered during tissue injury. Blood coagulation in vivo is influenced not only by the intrinsic and extrinsic coagulation pathways but also by dynamic interactions among vascular tissues, inflammatory cells, extracellular matrix components, and local biochemical mediators [20,21].

Furthermore, successful topical hemostatic materials should not only accelerate clot formation but also promote favorable tissue responses after implantation. Following hemostasis, the implanted scaffold is expected to undergo gradual biodegradation while minimizing excessive inflammatory reactions and supporting normal wound healing [16,17]. Therefore, evaluation of bleeding control, inflammatory cell infiltration, sponge degradation, and tissue integration under in vivo conditions represents an essential step in determining the translational potential of developed absorbable hemostatic biomaterials [22].

Based on these considerations, the optimized formulation (GC75) was subsequently selected for in vivo evaluation using a rat tooth extraction model. This experimental model provides a clinically relevant platform to investigate not only the immediate hemostatic efficacy of the scaffold but also its interaction with surrounding tissues during the early phases of wound healing. The following section therefore examines whether the favorable physicochemical characteristics and in vitro biological performance observed for GC75 can be manifested into effective hemostasis and acceptable tissue responses under physiological conditions.

### 3.3. In Vivo Evaluation of Hemostatic Efficacy and Host Tissue Response

Although the in vitro characterization demonstrated favorable physicochemical properties and promising hemostatic performance, the biological behavior of an implanted hemostatic biomaterial can only be fully evaluated under physiological conditions. Following implantation, the scaffold interacts simultaneously with blood components, inflammatory cells, extracellular matrix proteins, and host tissues, resulting in biological responses that cannot be completely reproduced by in vitro assays alone. Therefore, the rat tooth extraction model was employed to validate the hemostatic efficacy, tissue response, biodegradation behavior, and systemic biosafety of the optimized formulation (GC75) under clinically relevant conditions. This sequential evaluation represents an important step in translating the favorable in vitro performance of the composite sponge toward potential application as an absorbable topical hemostatic material [3,4,23].

The bleeding time results demonstrated that GC75 significantly reduced the time required to achieve complete hemostasis compared with the untreated negative control, while exhibiting a hemostatic performance comparable with that of the commercial gelatin sponge (Curaspon^®^). Moreover, the duration required to achieve hemostasis remained relatively consistent throughout the observation period, indicating that the effectiveness of the scaffold was maintained during the early postoperative phase. These findings are consistent with the in vitro coagulation assays discussed previously, in which GC75 exhibited the lowest blood clotting index, accelerated blood clotting time, and enhanced fibrin generation. Collectively, these observations suggest that the favorable physicochemical characteristics of GC75—including its interconnected porous architecture, high swelling capacity, and balanced degradation profile—facilitated rapid blood absorption and local concentration of coagulation components at the extraction socket [3,12,20].

Histological evaluation demonstrated significant differences in polymorphonuclear neutrophil (PMN) infiltration among the experimental groups on postoperative days 1 and 7, whereas no significant difference was observed on day 3. Specifically, GC75 exhibited higher PMN infiltration than the negative control on day 1 but did not differ significantly from Curaspon^®^, indicating that the initial inflammatory response induced by the composite scaffold was comparable to that elicited by the commercial hemostatic material. Importantly, these findings should not be interpreted as evidence of poor biocompatibility. Neutrophil recruitment represents the earliest stage of the normal host response to tissue injury and implanted biomaterials, contributing to wound debridement, microbial clearance, and initiation of subsequent tissue repair [16,17].

The persistence of PMN infiltration during the early postoperative period should be interpreted within the normal temporal sequence of wound healing rather than as an indication of excessive inflammation. Following tooth extraction, acute inflammatory cell recruitment is essential for removal of damaged tissue and preparation of the wound site before macrophage-mediated resolution and fibroblast proliferation occur [17]. Consequently, the inflammatory profile observed in the GC75 group appears compatible with physiological healing rather than adverse foreign-body reactions. Similar inflammatory patterns have been reported for biodegradable gelatin–chitosan scaffolds, in which transient neutrophil infiltration is followed by progressive tissue remodeling without evidence of chronic inflammatory responses [5,16].

The in vivo degradation findings further support this interpretation. Histopathological examination demonstrated that biomaterial residues remained detectable in a proportion of specimens until postoperative day 7, indicating that GC75 was not degraded immediately after implantation but instead persisted during the early stages of wound healing. Although descriptive analysis suggested a greater proportion of degraded specimens on postoperative days 1 and 7, statistical analysis did not identify significant differences among the experimental groups. These observations suggest that the degradation profile of GC75 was sufficiently controlled to maintain temporary structural support throughout the critical early phase of tissue repair.

Evaluation of hematological and blood biochemical parameters further demonstrated that implantation of GC75 did not induce detectable systemic toxicity during the observation period. Although statistical analyses identified variations associated with treatment groups or observation time for several hematological and biochemical variables, subsequent pairwise comparisons revealed no consistent significant differences between GC75 and either the negative or positive control groups. Similarly, serum AST, ALT, urea, and creatinine values remained within comparable ranges throughout the experimental period, with no progressive increase suggestive of hepatic or renal dysfunction.

Assessment of systemic biosafety is an essential component in the preclinical development of implantable hemostatic biomaterials because degradation products may enter the systemic circulation following implantation. According to ISO 10993-11, biochemical markers such as AST, ALT, urea, and creatinine are widely accepted indicators for evaluating potential hepatic and renal toxicity associated with implanted medical devices [17]. The absence of consistent alterations in these parameters indicates that the degradation products generated from the gelatin–chitosan composite were well tolerated under the experimental conditions. These findings are consistent with previous investigations demonstrating that both gelatin and chitosan exhibit favorable biosafety profiles because they are biodegradable natural polymers whose degradation products are readily metabolized or eliminated through normal physiological pathways [5,16].

The in vivo findings confirm that the physicochemical characterization and in vitro biological evaluation presented in the preceding sections. The ability of GC75 to achieve rapid bleeding control comparable to a commercial gelatin sponge, while maintaining physiological inflammatory responses, controlled biodegradation, and the absence of detectable systemic toxicity, supports its potential as a biodegradable topical hemostatic material for oral surgical applications. These results indicate that optimization of the gelatin-to-chitosan ratio not only improved the structural characteristics of the scaffold but also translated into favorable biological performance under physiological conditions, thereby strengthening the translational potential of this fish-derived biomaterial.

## 4. Materials and Methods

**Figure 10:**
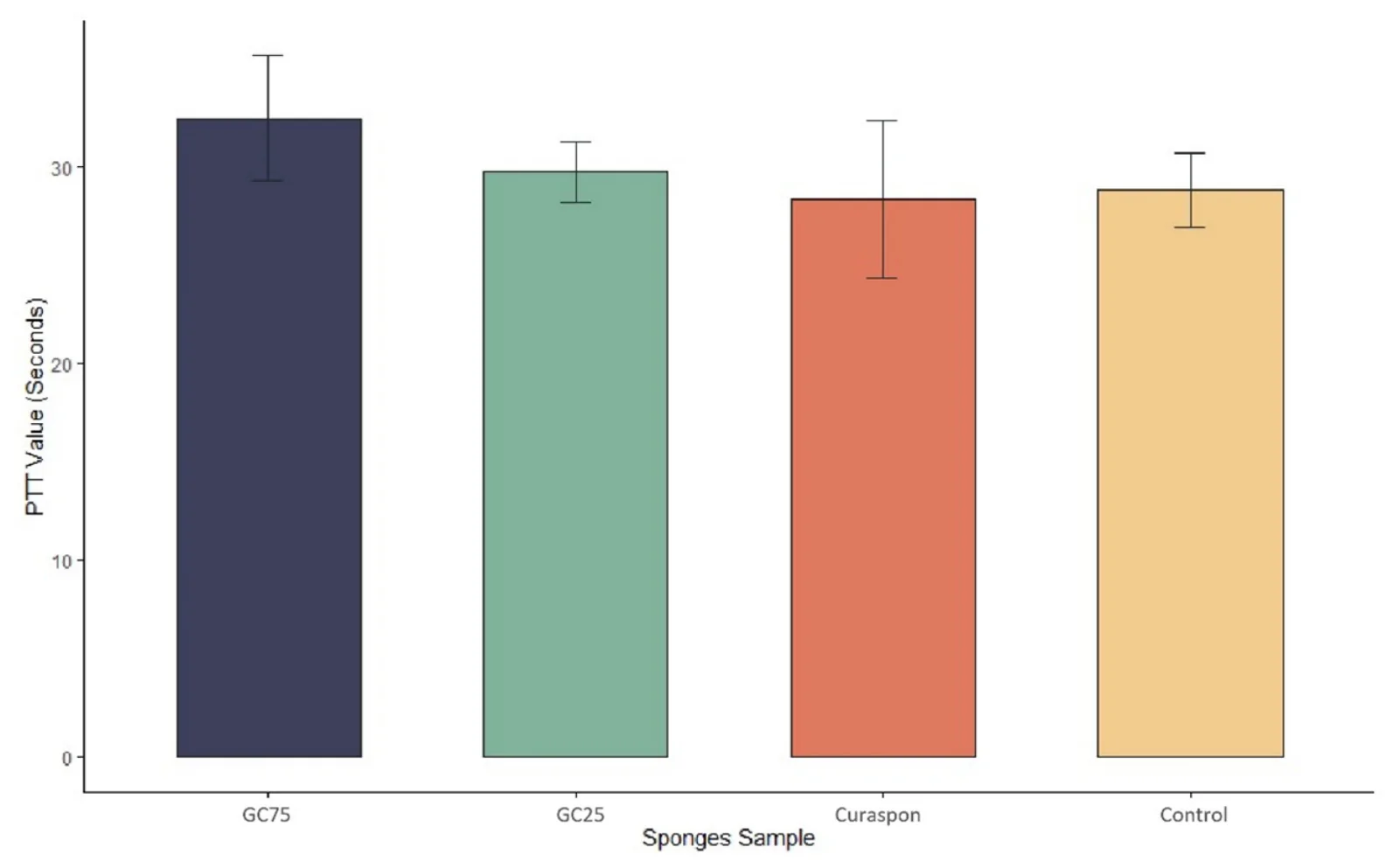
PTT values of GC75, GC25, Curaspon^®^, and control samples.

**Figure 11:**
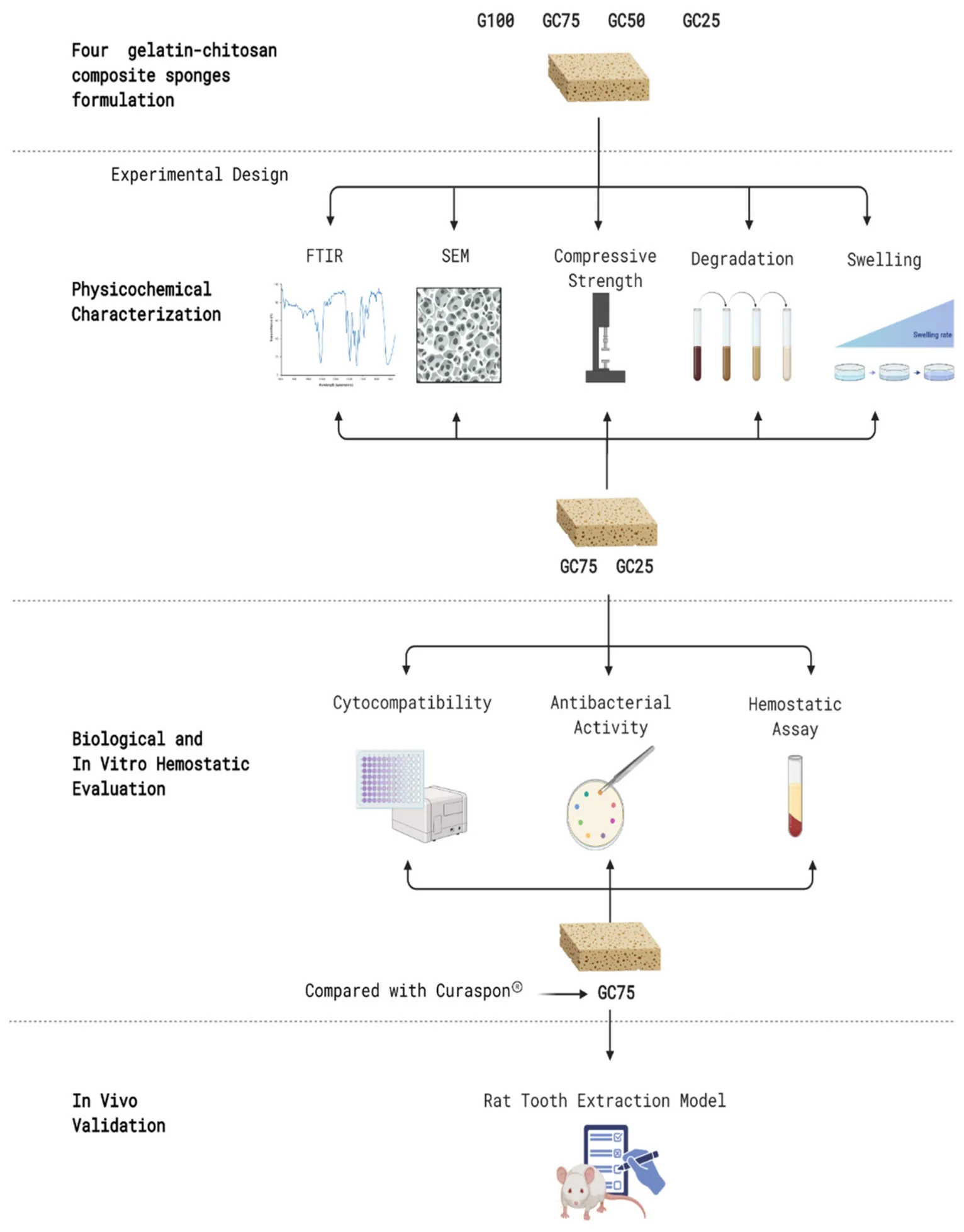
Schematic overview of the experimental design employed in this study. Created using https://BioRender.com.

The influence of gelatin–chitosan composition on scaffold characteristics and hemostatic performance was systematically investigated using a stepwise experimental design (Figure 10 and Figure 11). Four sponge formulations with different gelatin-to-chitosan ratios were fabricated and subjected to comprehensive physicochemical characterization. Based on these results, the two most potential formulations were selected for subsequent biological and in vitro hemostatic evaluations. The formulation exhibiting the best overall performance was further validated by comparison with a commercial gelatin sponge in an in vivo tooth extraction model.

### 4.1. Materials

Fish skin gelatin (Type A; average molecular weight approximately 200 kDa) extracted from Pangasius sp. was obtained from the Faculty of Fisheries and Marine Sciences, IPB University, Indonesia. Chitosan powder with a degree of deacetylation greater than 85% was supplied by the National Research and Innovation Agency (BRIN), Indonesia. Phosphate-buffered saline (PBS) was used for the swelling and in vitro degradation studies. Acetic acid, sodium hydroxide (NaOH), and ethanol were used during sponge fabrication. All chemicals were of analytical grade and were used without further purification. Addition of both chitosan and calcium acetate to fish gelatin scaffolds had reduced biodegradation rate in addition to increasing the mechanical features, which was suitable for biomedical applications [24].

For the in vitro coagulation assays, tissue thromboplastin (rabbit brain extract), porcellanite (0.4%), and calcium chloride (CaCl_2_) were obtained from MD Pacific Biotechnology Co., Ltd. (Tianjin, China). A commercially available absorbable gelatin sponge (Curaspon^®^) was used as the reference material during the comparative hemostatic evaluation.

### 4.2. Preparation of Gelatin–Chitosan Composite Sponges

Gelatin–chitosan composite sponges were fabricated by freeze-drying using four formulations with gelatin-to-chitosan weight ratios of 100:0 (G100), 75:25 (GC75), 50:50 (GC50), and 25:75 (GC25) to evaluate the effect of polymer composition on scaffold architecture and physicochemical properties. The total polymer content was fixed at 2 g for all formulations. Each formulation was dissolved in 50 mL of 1% (*v*/*v*) aqueous acetic acid and homogenized using a magnetic stirrer for 2 h to obtain a homogeneous polymer solution.

The homogeneous polymer solutions were poured into cubic molds (1.3 × 1.3 × 1.3 cm^3^) and frozen at −20 °C to form solid matrices. The frozen samples were subsequently immersed in a neutralization solution consisting of 1 M NaOH and ethanol at a ratio of 1:3 (total volume 100 mL) for 30 min. The samples were then rinsed thoroughly with distilled water until neutral pH was achieved, as confirmed using litmus paper. Following neutralization, the samples were refrozen at −20 °C before lyophilization.

**Figure 12:**
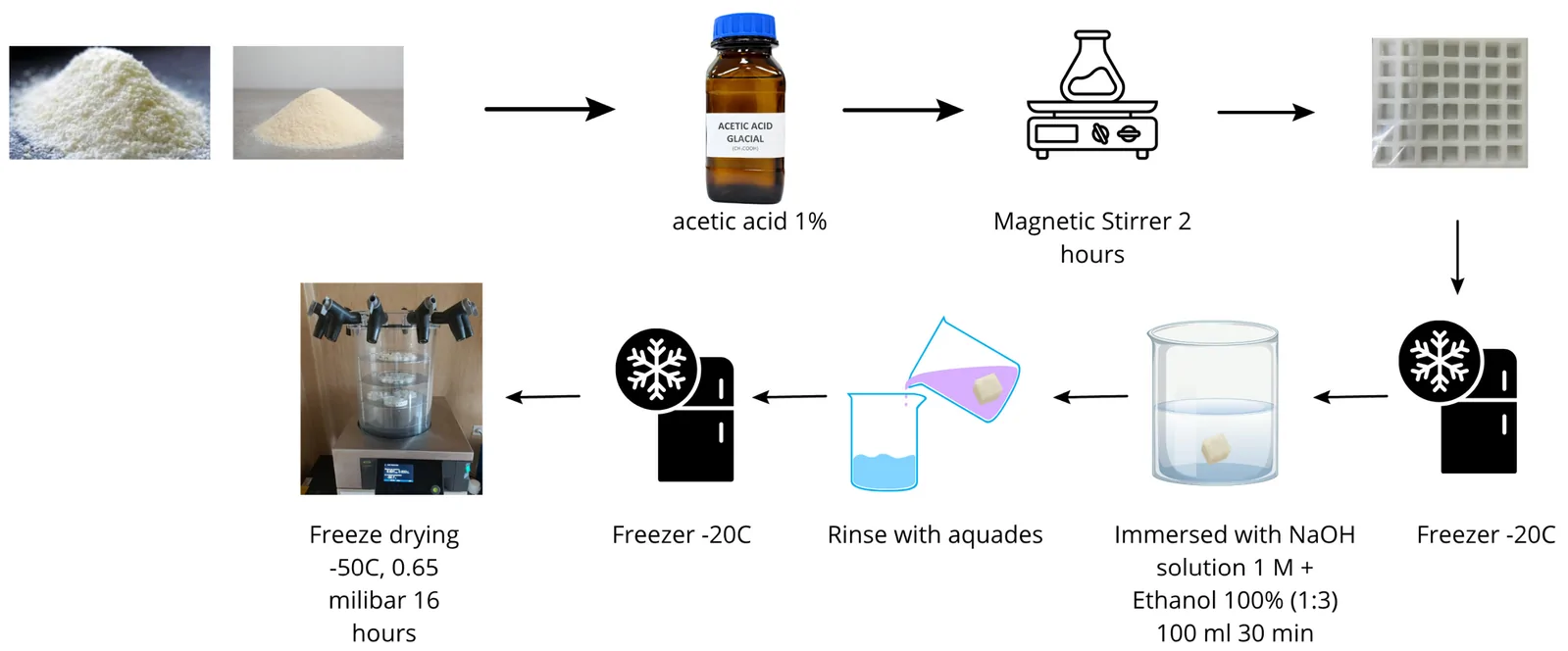
Schematic illustration of the fabrication process of gelatin–chitosan composite sponges using the freeze-drying technique. The process involved polymer dissolution, homogenization, molding, freezing, alkali neutralization, washing, refreezing, and lyophilization to obtain porous composite sponges.

Lyophilization was performed using a Lyovapor L-200 freeze dryer (Büchi, Flawil, Switzerland) at −50 °C and 0.65 mbar for 16 h to obtain porous gelatin–chitosan composite sponges. The prepared sponges were stored under appropriate conditions until physicochemical characterization, biological evaluation, in vitro hemostatic assessment, and in vivo validation. The fabrication process is illustrated in Figure 12.

### 4.3. Physicochemical Characterization

#### 4.3.1. FTIR

FTIR was performed to identify the characteristic functional groups of the gelatin–chitosan composite sponges and to evaluate the chemical interactions between the constituent polymers following the freeze-drying process. In drug delivery, Almukainzi et al. [25] used FTIR to analyze chitosan/hesperidin nanoparticles, identifying amine I NH-H bending (1600 cm^−1^), amide II carbonyl (1670 cm^−1^), p=O interactions, OH stretching (2923.09 cm^−1^), carbonyl groups (1644.35 cm^−1^), and aromatic rings (1517.22 cm^−1^). FTIR spectra of all formulations (G100, GC75, GC50, and GC25) were recorded over the wavenumber range of 600–4000 cm^−1^ [25]. FTIR has proven invaluable in tissue engineering and biopolymer research, providing critical insights into the structural and functional properties of polysaccharides across a wide spectrum of scientific and industrial application [26]. The resulting spectra were analyzed by comparing the characteristic absorption bands of gelatin and chitosan to assess the preservation of their chemical structures and the presence of intermolecular interactions within the composite scaffold.

#### 4.3.2. SEM

The surface morphology and porous microstructure of the fabricated gelatin–chitosan composite sponges were examined using scanning electron microscopy (SEM, Thermo, Waltham, MA, USA). All formulations (G100, GC75, GC50, and GC25) were observed to evaluate the effect of gelatin–chitosan composition on pore morphology and scaffold architecture. Representative SEM micrographs acquired at 100× magnification were subjected to quantitative image analysis using ImageJ 1.48 software. For pore-area analysis, images were calibrated using the scale bar (346 pixels = 1 mm), and an identical region of interest (1024 × 884 pixels) was analyzed using Otsu thresholding to determine the two-dimensional pore-area fraction (%). In addition, pore dimensions were determined from ten morphometric measurements obtained from representative SEM micrographs for each formulation. Mean, standard deviation, median, range, and pore-size distributions were descriptively determined. Pore sizes were categorized as <100, 100–200, 200–300, 300–400, and >400 µm.

#### 4.3.3. Compressive Strength Test

The compressive mechanical properties of the fabricated gelatin–chitosan composite sponges were evaluated to determine the influence of gelatin–chitosan composition on scaffold mechanical stability. All formulations (G100, GC75, GC50, and GC25) were tested using a Universal Testing Machine (H5K-S, Hounsfield Test Equipment Ltd., Surrey, UK).

Prior to testing, sponge specimens were cut into dimensions of 10 × 10 × 2 mm^3^ and mounted between the testing fixtures. The samples were subjected to compression at a constant crosshead speed of 1 mm min^−1^ until failure, and the maximum compressive stress was recorded for each specimen. Three independent specimens were tested for each formulation, and the results were used to compare the mechanical performance of the different gelatin–chitosan compositions.

#### 4.3.4. Swelling Capacity

The swelling capacity of the fabricated gelatin–chitosan composite sponges were evaluated to determine their fluid absorption ability under physiological conditions. All formulations (G100, GC75, GC50, and GC25) were tested by immersing the dried sponge samples in 5 mL of phosphate-buffered saline (PBS) at 37 °C. Prior to immersion, each sample was weighed to obtain its initial dry weight (*W_dry_*).

At predetermined time intervals (1, 5, 10, and 30 min), the samples were removed from the PBS solution, gently blotted with filter paper to remove excess surface liquid, and immediately weighed to obtain the swollen weight (*W_w_*). The swelling capacity was calculated using the following equation:

(1) $$\mathrm{Swelling Capacity}\left(\%\right)=\frac{W_{w}-W_{\mathrm{dry}}}{W_{\mathrm{dry}}}\times 100$$

where *W_dry_* is the initial dry weight and *W_w_* is the swollen weight at each observation time. Three independent specimens were tested for each formulation at every time point, and the results were used to compare the fluid absorption behavior of the different gelatin–chitosan composite sponges.

#### 4.3.5. In Vitro Degradation

The in vitro degradation behavior of the fabricated gelatin–chitosan composite sponges were evaluated to determine their structural stability under physiological conditions. All formulations (G100, GC75, GC50, and GC25) were initially weighed to obtain the initial dry weight (*W*_0_). Each sponge sample was then placed in a 50 mL conical tube containing 25 mL of phosphate-buffered saline (PBS) and incubated at 37 °C for 7 days.

The samples were removed from the PBS solution on days 3, 5, and 7 days, gently blotted with filter paper to remove excess surface liquid, and weighed to obtain the remaining weight (*W_t_*). The degradation percentage was calculated using the following equation:

(2) $$\mathrm{Degradation}\left(\%\right)=\frac{W_{0}-W_{t}}{W_{0}}\times 100$$

where *W*_0_ is the initial dry weight and *W_t_* is the remaining weight at each observation time. Three independent specimens were analyzed for each formulation, and the degradation profiles were compared to evaluate the influence of gelatin–chitosan composition on scaffold stability under physiological conditions.

### 4.4. Biological Evaluation

The biological performance of the selected gelatin–chitosan composite sponges were evaluated to assess their cytocompatibility and antibacterial properties prior to in vitro hemostatic and in vivo evaluations. Based on the physicochemical characterization results, only the two most promising formulations (GC75 and GC25) were selected for biological assessment.

#### 4.4.1. Cytocompatibility

The cytocompatibility of the selected gelatin–chitosan composite sponges (GC75 and GC25) were evaluated using a standard 3-(4,5-dimethylthiazol-2-yl)-2,5-diphenyltetrazolium bromide (MTT) assay with human gingival fibroblasts (HGFs). The cells were obtained from Cell Line Service Co. (Dainippon, Osaka, Japan) and seeded into 96-well plates at a density of 1 × 10^4^ cells per well. The cultures were incubated for 24 h at 37 °C in a humidified atmosphere containing 5% CO_2_ to allow cell attachment.

Following incubation, the culture medium was replaced with fresh medium containing the test samples. GC75 and GC25 were evaluated, while untreated cells served as the negative control (100% cell viability) and the commercial gelatin sponge (Curaspon^®^) served as the positive control. Each experimental condition was tested in six replicates and performed in duplicate experiments (*n* = 12). After 24 h of exposure, the treatment medium was removed, and the cells were gently washed with phosphate-buffered saline (PBS).

Subsequently, MTT solution (0.5 mg mL^−1^) was added to each well and incubated for 3–4 h at 37 °C to allow intracellular reduction of MTT into insoluble formazan crystals. The MTT solution was then discarded, and the crystals were dissolved using *dimethyl sulfoxide (DMSO).* Absorbance was measured at 570 nm using a microplate reader. Cell viability was calculated according to the following equation:

(3) $$\mathrm{Cell viability}\left(\%\right)=\frac{\mathrm{Absorbance of sample}}{\mathrm{Absorbance of negative control}}\times 100$$

where the absorbance of the untreated control cells was defined as 100% cell viability.

#### 4.4.2. Antibacterial Activity Assay

The antibacterial activity of the selected gelatin–chitosan composite sponges (GC75 and GC25) were evaluated using the agar well diffusion method against *Staphylococcus aureus*. Sterile nutrient agar plates were prepared, and a bacterial suspension was uniformly inoculated onto the agar surface using a semi-solid overlay technique. Wells were subsequently created in the agar using a sterile cork borer.

Approximately 50 µL of each test sample was introduced into the respective agar well. The commercial gelatin sponge (Curaspon^®^) was included as the reference material. The inoculated plates were incubated at 37 °C for 24 h, after which antibacterial activity was assessed by measuring the diameter of the inhibition zone surrounding each well. The absence of a visible inhibition zone was interpreted as the absence of detectable antibacterial activity under the experimental conditions.

### 4.5. In Vitro Hemostatic Assay

The in vitro hemostatic performance of the selected gelatin–chitosan composite sponges (GC75 and GC25) were evaluated using complementary coagulation assays to assess different aspects of blood–material interactions. The commercial gelatin sponge (Curaspon^®^) was included as the reference material. The evaluation included BCI, BCT, fibrin generation assay, PT, and PTT. Fresh human whole blood and platelet-poor plasma (PPP) were collected from healthy adult volunteers after obtaining written informed consent. Fresh human blood and PPP were subsequently prepared according to the respective assay requirements for each hemostatic assay.

#### 4.5.1. BCI

The blood clotting ability of the hemostatic sponges was evaluated using BCI method [27]. Sponge samples weighing 10 mg were placed into test tubes and mixed with 200 µL of fresh citrated blood. The control group consisted of blood without sponge material. The mixtures were incubated at 37 °C for 5 min.

Then, 10 mL of deionized water was added to lyse unbound red blood cells. The solution was gently mixed at 30 rpm for 10 min. Absorbance was measured at 490 nm using a microplate reader. The *BCI* value was calculated using the following equation:

(4) $$\mathrm{BCI}\left(\%\right)=\left(\frac{A_{s}}{A_{c}}\right)\times 100$$

where *As* represents the absorbance of the sample and *Ac* represents the absorbance of the control.

#### 4.5.2. BCT

Blood clotting time was measured to evaluate the coagulation acceleration ability of the hemostatic sponges. Sponge samples (1 cm × 1 cm × 1 cm) were placed in test tubes and mixed with 2 mL of fresh whole blood. The samples were incubated at 37 °C. Blood clotting time was recorded using a chronometer from the moment blood was added until complete clot formation occurred. Complete coagulation was defined as the absence of visible liquid blood in the tube. Blood without sponge material served as the control group.

#### 4.5.3. PTT Assay

Partial thromboplastin time was measured to evaluate the intrinsic coagulation pathway. Sponge samples were mixed with 100 µL plasma and 100 µL porcellanite reagent and incubated at 37 °C for 5 min. After incubation, one bead and 100 µL CaCl_2_ were added to initiate clot formation. The coagulation analyzer automatically measured the clotting time. Results were compared with the standard reference range (21–38 s).

#### 4.5.4. PT Assay

Prothrombin time was measured using a TS-6000 coagulation analyzer. A volume of 100 µL plasma was placed into the cuvette, followed by insertion of the sponge sample (2.5 × 10 × 10 mm^3^). The mixture was incubated at 37 °C for 3 min. Thromboplastin reagent of 200 µL was added to initiate the extrinsic coagulation pathway. The coagulation analyzer automatically recorded the clotting time. Results were interpreted according to the kit reference range (10–15 s).

#### 4.5.5. Fibrin Generation Assay

Fibrin generation was evaluated using a turbidity-based assay. Sponge samples (5 mg) were placed in a 96-well microplate and mixed with 85 µL of platelet-poor plasma. The plate was incubated at 37 °C for 5 min. After incubation, 10 µL of CaCl_2_ solution was added to initiate the coagulation cascade. Absorbance was measured at 405 nm every minute for 60 min using a microplate reader. Fibrin initiation time was defined as the time point when the absorbance increased by 10% above the baseline value. The maximum slope of the turbidity curve was used to determine fibrin generation rate.

### 4.6. In Vivo Evaluation

The in vivo performance of the selected gelatin–chitosan composite sponge (GC75) was evaluated using a rat tooth extraction model to assess its hemostatic efficacy, biodegradation behavior, inflammatory response, and systemic safety. The experimental evaluation included bleeding time measurement, histopathological analysis, assessment of biomaterial degradation, polymorphonuclear neutrophil (PMN) infiltration, and hematological and serum biochemical analyses.

#### 4.6.1. Animal Model

Male Sprague Dawley rats weighing 250–300 g was used for the in vivo evaluation. Before the experiment, the animals were acclimatized for 7 days under standard laboratory conditions. Rats were housed in polycarbonate cages (2–3 animals per cage) under controlled environmental conditions (22 ± 2 °C, 50–60% relative humidity, and a 12 h light/12 h dark cycle) with free access to standard laboratory chow and water throughout the study. Animals were monitored daily during the acclimatization period, and only healthy animals without clinical abnormalities were included in the experiment.

#### 4.6.2. Rat Tooth Extraction Model

The animals were randomly allocated into three experimental groups: (1) treatment group receiving the selected gelatin–chitosan composite sponge (GC75), (2) positive control group receiving the commercially available absorbable gelatin sponge (Curaspon^®^), and (3) negative control group without hemostatic material. Each group was further subdivided according to the postoperative observation periods (days 1, 3, and 7).

General anesthesia was induced by intraperitoneal administration of ketamine (30 mg kg^−1^) combined with xylazine. Following adequate anesthesia, a standardized tooth extraction was performed to create an extraction socket. Bleeding was allowed to continue freely for 5 s, then GC75 sponge was immediately applied to the extraction socket in the treatment and positive control groups, whereas no material was applied in the negative control group. Gentle compression was applied using sterile forceps until complete hemostasis was achieved.

Hemostatic efficacy was evaluated by measuring the bleeding time, defined as the interval between application of the test material and complete cessation of bleeding. To verify complete hemostasis, the material was carefully lifted from the extraction socket every 10 s. If active bleeding persisted, the material was repositioned and gentle compression was continued until bleeding completely ceased. The total bleeding time was recorded for each animal.

After hemostasis was achieved, the extraction socket was closed using 3–0 silk sutures. Animals received postoperative enrofloxacin as antibiotic prophylaxis and meloxicam (Melovem^®^) for analgesia before being returned to their cages. Animals were euthanized on postoperative days 1, 3, and 7, the extraction socket and surrounding tissues were harvested for histopathological evaluation. Blood samples were collected on postoperative day 7 for hematological and serum biochemical analyses.

#### 4.6.3. Histopathological Evaluation

Following euthanasia on postoperative days 1, 3, and 7, the extraction socket together with the surrounding alveolar tissue was harvested and immediately fixed in 10% neutral buffered formalin. The specimens were subsequently processed using standard histological procedures, embedded in paraffin, sectioned, and stained with hematoxylin and eosin (H&E) for microscopic examination.

Histopathological evaluation was performed to assess the tissue response following implantation of the hemostatic material. The examined parameters included polymorphonuclear neutrophil (PMN) infiltration, biomaterial degradation, and the overall morphology of the healing tissue. PMN infiltration was evaluated as an indicator of the acute inflammatory response, whereas biomaterial degradation was assessed by determining the presence or absence of residual sponge material within the extraction socket.

Histological observations were performed at each postoperative time point (days 1, 3, and 7) and compared among the treatment, positive control, and negative control groups to evaluate the biological response to the implanted materials.

#### 4.6.4. Hematological and Serum Biochemical Analysis

Blood samples were collected from all experimental groups on postoperative day 7 to evaluate the systemic biocompatibility of the implanted materials. Hematological analysis included total leukocyte count and differential leukocyte counts, including neutrophils, lymphocytes, and monocytes, to assess the systemic inflammatory response.

Serum biochemical analysis was performed to evaluate possible hepatic and renal toxicity following implantation of the hemostatic materials. The evaluated biochemical parameters included aspartate aminotransferase (AST), alanine aminotransferase (ALT), urea, and creatinine. Hematological and biochemical parameters were compared among the treatment, positive control, and negative control groups to assess the systemic safety of the implanted gelatin–chitosan composite sponges.

### 4.7. Statistical Analysis

All experiments were performed in triplicate, and data are presented as mean ± standard deviation. Statistical analyzes were performed using IBM SPSS Statistics version 23 after assessment of data normality. The physicochemical properties of the sponges were analyzed to compare the effects of different formulations. BCI data were analyzed using mixed ANOVA, whereas BCT data were analyzed using Kaplan–Meier survival analysis. The fibrin generation assay was analyzed using the Kruskal–Wallis test followed by Dunn’s post hoc test, while PT and PTT values were compared using the Mann–Whitney U test. Data visualization was performed using R software version 4.3.3. Statistical significance was set at *p* < 0.05.

## 5. Conclusions

This study successfully developed fish gelatin–chitosan composite sponges using a freeze-drying technique and demonstrated that the gelatin-to-chitosan ratio plays a critical role in determining the physicochemical and biological performance of the scaffold. Among the investigated formulations, GC75 exhibited the most balanced characteristics, including a well-interconnected porous microstructure, favorable swelling capacity, controlled degradation behavior, and adequate mechanical stability. These physicochemical properties were consistently associated with acceptable cytocompatibility, efficient in vitro hemostatic performance, and effective bleeding control in the rat tooth extraction model, while maintaining physiological inflammatory responses and showing no evidence of systemic toxicity based on hematological and blood biochemical analyses.

The integration of physicochemical characterization, in vitro biological evaluation, and in vivo validation provides comprehensive evidence supporting the potential of the developed composite sponge as a biodegradable topical hemostatic biomaterial. The findings suggest that combining fish gelatin with chitosan not only improves scaffold architecture and blood absorption capacity but also promotes local hemostasis without compromising biocompatibility or host tissue responses. Therefore, the present study establishes a scientific basis for the development of fish-derived gelatin–chitosan composite sponges as a promising alternative to mammalian-derived absorbable hemostatic materials for oral and maxillofacial surgical applications.

Several limitations should nevertheless be acknowledged. The present study focused primarily on the evaluation of physicochemical characteristics, hemostatic performance, and early biological responses, In addition, the Blood Clotting Index assay was performed using citrated whole blood under standardized experimental conditions and therefore represents a comparative assessment of blood–material interactions rather than a complete stimulation of the physiological coagulation cascade. Consequently, the long-term degradation behavior, detailed cellular mechanisms involved in blood–material interactions, and functional tissue regeneration following complete scaffold resorption remain to be elucidated. In addition, the in vivo evaluation was performed using a small-animal tooth extraction model, which may not fully represent the clinical conditions encountered in humans. Future studies should therefore investigate the long-term biological performance of the scaffold, elucidate the molecular mechanisms underlying its hemostatic activity, and validate its efficacy and safety in larger animal models before clinical translation.

## Figures and Tables

**Table 1:** Quantitative pore characteristics of freeze-dried gelatin–chitosan sponges based on SEM image analysis.

Formulation	2D Pore Area Fraction (%)	Mean Pore Size ± SD (µm)	Median Pore Size (µm)	Pore-Size Range (µm)
G100	62.70	64.86 ± 28.00	52.28	38.33–127.10
GC75	63.86	220.16 ± 122.25	201.45	112.1–525.60
GC50	67.24	231.94 ± 107.31	219.00	75.60–422.50
GC25	56.69	193.93 ± 132.14	160.30	51.56–444.60

Note: The 2D pore area fraction was determined using ImageJ after scale calibration and Otsu thresholding. Pore-size measurements were obtained from representative SEM micrographs (*n* = 10 measurements per formulation). Values for pore size are descriptive morphometric measurements and were not treated as independent experimental replicates.

**Table 2:** Swelling capacity (%) of G100, GC75, GC50, and GC25 following immersion in PBS for 1, 5, 10, and 30 min.

Formulation	1 min	5 min	10 min	30 min
G100	149.68 ± 44.94	Dissolved	Dissolved	Dissolved
GC50	586.34 ± 50.11	479.51 ± 91.67	614.67 ± 37.94	612.66 ± 37.04
GC25	740.92 ± 30.54	252.50 ± 56.81	569.73 ± 85.16	532.20 ± 78.49
GC75	586.40 ± 41.75	1115.22 ± 137.94	619.47 ± 36.71	600.07± 43.26

**Table 3:** In vitro degradation profiles of G100, GC75, GC50, and GC25 following incubation in PBS at 37 °C.

Formulation	Day 3 (mg)	Day 5 (mg)	Day 7 (mg)	Degradation Rate
G100	0	0	0	Complete degradation by day 3
GC50	15.63± 7.28	19.20 ± 5.33	20.64± 5.68	Gradual (~30% by day 14)
GC25	9.76 ± 4.04	12.96± 2.69	15.81 ± 2.76	Slow (~18% by day 14)
GC75	33.88± 7.55	38.72 ± 5.70	48.73 ± 5.07	Moderate-fast (~51% by day 14)

**Table 4:** BCI results.

Sample	M ± SD	*p*-Value
GC75	91.42 ± 4.06	<0.001
GC25	92.54 ± 4.95	
Curaspon^®^	100.97 ± 1.75	

M = median; SD = standard deviation.

**Table 5:** BCT analysis using survival analysis (Kaplan–Meier).

Sample	M ± SD	Log-Rank Test		
		*χ* ^2^	df	*p*
GC75	8.61 ± 0.29	20.25	1	0.001
GC25	11.99 ± 2.07			
Curaspon^®^	9.87 ± 0.44			
Control	13.87 ± 0.6			

*p* < 0.001; M = mean; SD = standard deviation; df = degrees of freedom; χ^2^ = chi-square value.

**Table 6:** Summary of in vitro hemostatic performance of the tested materials.

Assay	GC75	GC25	Curaspon^®^	Interpretation
BCI	91.42 ± 4.06	92.54 ± 4.95	100.97 ± 1.75	GC75 exhibits the lowest BCI value, indicating the most efficient clot formation among the tested materials
BCT (min)	8.61 ± 0.29	11.99 ± 2.07	9.87 ± 0.44	GC75 achieved the shortest clotting time, demonstrating accelerated coagulation
Fibrin Generation	Highest	Moderate	Lower than GC75	GC75 promoted the greatest fibrin formation, suggesting enhanced fibrin polymerization
PT (s)	12.8 ± 2.52	14.57 ± 0.35	15.7 ± 3.55	All formulations maintained PT values within the physiological range, indicating no disruption of the extrinsic coagulation pathway
PTT (s)	32.43	29.73	28.30	No significant differences were observed among groups, indicating preservation of the intrinsic coagulation pathway

**Table 7:** Descriptive statistics of bleeding time according to experimental group and observation day.

Group	Observation Day	*n*	Mean ± SD (s)	Median (s)
Negative control	Day-1	4	29.34 ± 1.83	29.53
	Day-3		31.07 ± 5.21	30.08
	Day-7		38.90 ± 10.74	34.80
Positif control	Day-1	4	19.83 ± 5.17	21.08
	Day-3		21.73 ± 3.95	21.64
	Day-7		24.58 ± 2.08	23.89
GC75	Day-1	4	20.25 ± 5.18	20.11
	Day-3		19.95 ± 4.44	18.76
	Day-7		20.22 ± 8.86	16.58

## Data Availability

The original contributions presented in this study are included in the article. Further inquiries can be directed to the corresponding author.

## Abbreviations

The following abbreviations are used in this manuscript:

G100	Gelatin 100%
GC75	Gelatin 75% Chitosan 25%
GC50	Gelatin 50% Chitosan 50%
GC25	Gelatin 25% Chitosan 75%
FTIR	Fourier Transform Infrared Spectroscopy
BCI	Blood Clotting Index
BCT	Blood Clotting Time
PT	Prothrombin Time
PTT	Partial Thromboplastin Time
